# Accessing New Phenanthroline–Oxazine Scaffolds as Copper‐Dependent DNA Damaging Probes

**DOI:** 10.1002/cbic.202500874

**Published:** 2026-04-17

**Authors:** Rebecca Lynn, Alex Gibney, Eva Delahunt, Stephen MacDonald‐Brown, Carlos Lence, Miles Kenny, Matt Allen, Ravil Khaybullin, Iva Lukac, Andrew Jordan, Andrew Kellett

**Affiliations:** ^1^ Research Ireland Centre for Pharmaceuticals School of Chemical Sciences Dublin City University Dublin Ireland; ^2^ Charnwood Discovery Charnwood Campus Loughborough UK

**Keywords:** amino acid, chemical nuclease, copper, DNA binding, oxazine

## Abstract

1,10‐Phenanthroline (phen) is a versatile ligand commonly used in coordination chemistry, with particular interest in its ability to promote DNA binding and damage when coordinated with transition metals. Structural modifications of phen modulate the recognition and reactivity of the associated complexes at the nucleic acid interface. A promising new modification of phen stems from the incorporation of amino acid derivatives at the 5‐ and 6‐positions leading to phenanthroline–oxazine scaffolds, which improve the pharmacokinetic and pharmacodynamic properties of resultant complexes. However, a limited number of phenanthroline–oxazine (PO) scaffolds have been reported and accessing new natural amino acid derivatives has, thus far, been synthetically intractable. Herein, we advance the synthetic conditions required for accessing new POs. We identified the type of amino acid substrate coupled with solvent selection and reaction temperature were the main influencing parameters for accessing new PO scaffolds. In total, eleven new PO ligands were successfully synthesized, purified, and characterized. The new scaffolds were then examined for their Cu‐dependent DNA binding and damaging profiles and we correlate structure–activity relationships to specific PO modifications.

## Graphical abstract

New phenanthroline–oxazine ligands derived from natural amino acids are synthesized through an optimized, predictive strategy. When coordinated to copper, these scaffolds display tunable DNA binding, groove selectivity, and oxidative cleavage activity, revealing clear structure activity relationships and expanding the toolbox of copper‐dependent artificial metallonucleases.

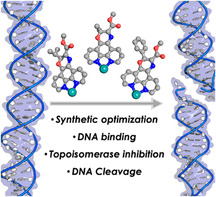

## 1 Introduction

1,10‐Phenanthroline (phen) is a versatile bidentate ligand that has attracted attention in a wide variety of applications, with a planar aromatic structure and nitrogen donors for metal ion coordination. Thus, phen has become an important ligand for chemists targeting metal complexes to DNA [1]. The rigid, planar structure of phen differentiates it from other classical bidentate chelates like 2,2′‐bipyridine (bpy), often allowing phen‐containing complexes to semi‐intercalate the DNA duplex by partially binding between DNA bases and forming *π*–*π* stacking interactions [1, 2]. Derivatization of phen has resulted in a variety of ligands that influence DNA recognition. For examples, ligands with extended aromatic structures, like dipyrido[3,2‐*a*:2′, 3′‐*c*]phenazine (dppz), have been found to greatly enhance the DNA binding properties of metal complexes, including copper [3]. Inspiration for studying this ligand stems from seminal work on the DNA intercalation properties of [Ru(dppz)(bpy)_2_]^2+^, also known as the “DNA light switch” complex due to its luminescent properties when bound with double‐stranded DNA (dsDNA) [4].

Synthetically, phen can be functionalized at various ring positions, with symmetric addition at the 2,9‐ and 5,6‐positions being most common, allowing further functionalization and tuning for specific applications [5]. Oxidation of the 2,9‐dimethyl derivative (neocuproine) can be used to install additional donors and access caging systems through intermediate dialdehydes, while that at the 5,6‐position provides a diketone (phendione) that can be used for Schiff base‐mediated extension of the aromatic surface such as in the case of dppz.

One of the most promising applications of phen‐based metal complexes is as artificial metallonucleases (AMNs). AMNs are transition metal complexes that oxidatively cleave DNA [6]. In 1979, Sigman identified that [Cu(phen)_2_]^2+^ inhibited DNA polymerase I and later established its role as an AMN [7, 8, 9, 10]. Since this discovery, various phen derivatives and complexes have been investigated as AMNs, resulting in a plethora of developmental agents and expanded interest in Cu‐based AMNs as a whole [3, 11, 12, 13, 14, 15, 16, 17, 18, 19]. While AMNs have continued to be applied to complex systems like gene editing strategies and targeting of noncanonical DNA structures, novel AMNs with tuned activities and activity profiles are of the utmost importance as they act as the central active group in such technologies [20, 21].

**FIGURE 1:**
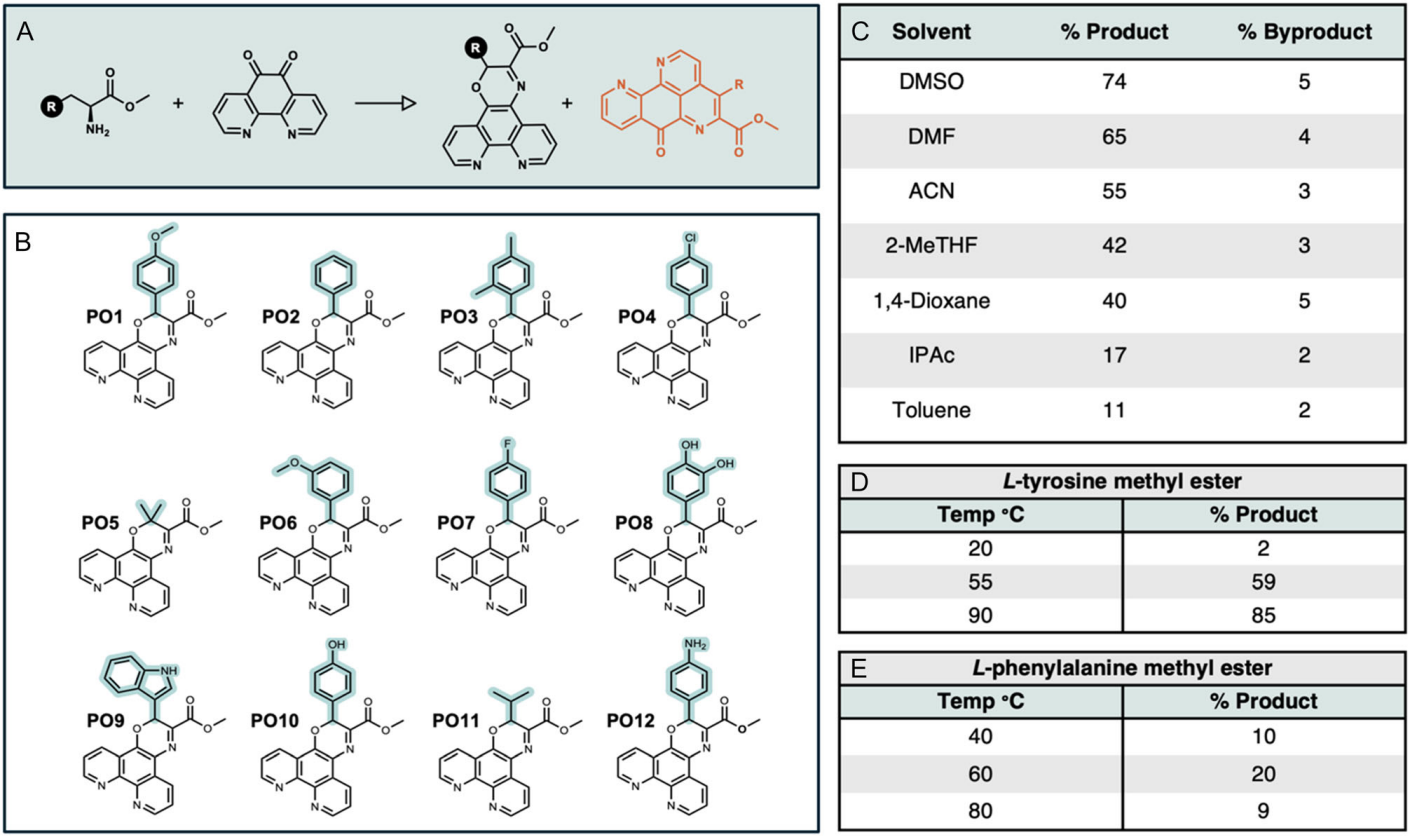
(A) The general reaction scheme of an amino acid methyl ester with phendione to generate the target PO ligand and an ascididemin by‐product (orange). (B) The structures of the 12 PO ligands synthesized in this study (note: PO10 is identical to the earlier reported PDT ligand reported by this group in 2013) [22]. (C) Solvent screening results in the reaction between *L*‐tyrosine methyl ester with phendione (DMSO = dimethyl sulfoxide; DMF = dimethylformamide; ACN = acetonitrile; 2‐MeTHF = 2‐methyltetrahydrofuran; IPAc = isopropyl acetate). (D) Temperature‐dependent product formation in the reaction of *L*‐tyrosine methyl ester with phendione. (E) Temperature‐dependent product formation in the reaction of *L*‐phenylalanine methyl ester with phendione.

In 2013, our group reported the coordination chemistry and atypical DNA binding activity of a new phenanthroline–oxazine (PO) ligand, named PDT, generated via the introduction of *L*‐tyrosine methyl ester to phendione through an oxazine bridge [22]. X‐ray structural analysis revealed that the tyrosine moiety in the PDT ligand is oriented orthogonally to the *N*, *N*‐phenanthroline plane, providing new opportunities for DNA recognition, and the resulting [Cu(PDT)_2_]^2+^ complex binds DNA through a strong, nonintercalative, surface, or groove‐associated mode distinct from the classical semi‐intercalation observed for [Cu(phen)_2_]^2+^. The synthetic approach was challenging and relatively low yielding (21%), which was later attributed by Ahmed et al. [23] to a competing reaction pathway that generated pyrido‐phenanthrolin‐7‐one compounds which are analogs of the marine alkaloid ascididemin (Figure 1A). The same work examined the effect of temperature and amino acid structure on the predominant reaction pathway, reporting that higher temperatures and electron withdrawing groups promoted the formation of the ascididemin analogs [23]. The work also showed that chirality within the PDT molecule was retained [22]. Herein, we report the first systematic study on the conditions required to access new organic PO scaffolds from *L*‐amino acid methyl esters and phendione. A number of factors, such as reagent ratio, base equivalents, time, temperature, and solvent, were examined. Further analysis on the substitution parameters (Hammett) was probed to identify routes most likely to yield new PO agents. In total, 11 new PO ligands were prepared and characterized (Figure 1B). We then examined the ability of the new ligands to recognize and damage dsDNA in the presence of copper. Both 1:1 and 1:2 stoichiometries where examined in situ with results showing high‐affinity binding, oxidative damage, and tuning of behavior toward intercalation.

## 2 Results and Discussion

### 2.1 Synthetic Studies

**FIGURE 2:**
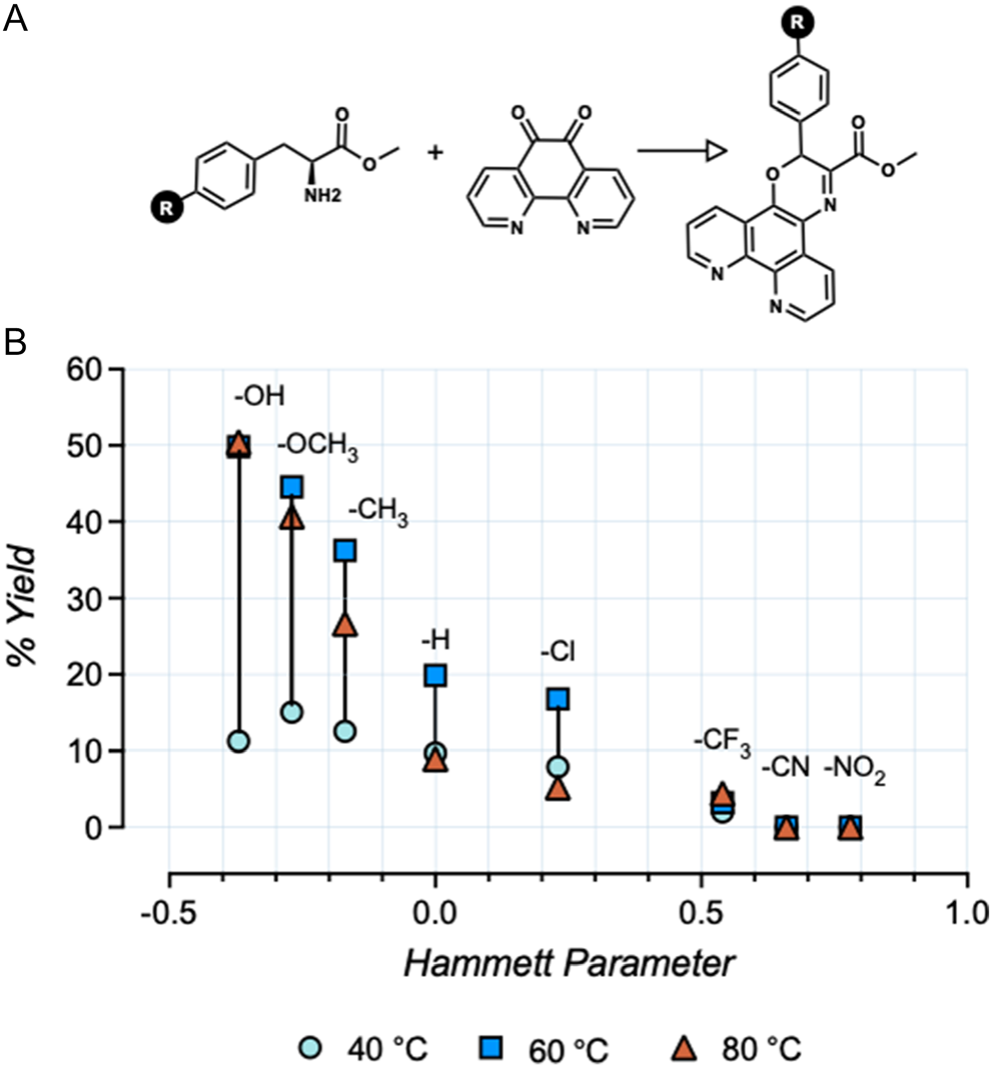
(A) The reaction scheme highlighting the differing *para‐*substituted groups on the methyl ester that reacts with phendione to generate the PO product. (B) Effect of *para‐*substituent‐associated Hammett parameter on the reaction efficiency at three temperatures.

A solvent screen was conducted following the general method established by Ahmed et al. [23] whereby phendione, *L*‐tyrosine methyl ester hydrochloride, 4‐methylmorpholine, and selected solvents were heated to 75°C for 24 h. Next, we measured the conversion of phendione to the target PO and the undesired ascididemin using electrospray ionization–mass spectrometry (ESI–MS) and found that DMSO gave the highest conversion to PO (Figure 1C). DMSO was therefore selected for subsequent reactions. We next investigated the effect of reactant ratios and temperature toward the formation of the desired PO product in the reaction of *L*‐tyrosine and *L*‐phenylalanine methyl esters (Figure 1D,E, and Table S1). Here, we found that reactant and base equivalents had no measurable impact on product formation but reaction temperature was significant. In the case of *L*‐tyrosine methyl ester, PO product formation had a strong linear correlation with higher temperatures, while for the phenylalanine reaction, a nonlinear relationship was found, with the highest percentage product forming at 60°C. Noting the varied PO conversions in both *L*‐tyrosine and *L*‐phenylalanine methyl esters reactions, together with the earlier findings of Ahmed et al., we next conducted synthetic analysis using a variety of *para*‐substituted phenylalanine analogs, covering a range of Hammett values that we theorized could aid in predicting successful product formation. Noting the earlier effects of reaction temperature in both *L*‐tyrosine and *L*‐phenylalanine methyl ester reactions, we conducted temperature‐dependent studies in this set of reactions (Figure 2).

Here, we found the Hammett parameter negatively correlated with PO product formation as –CF_3_, –CN, and –NO_2_ analogs yielded minimal product formation, while –OH, –OMe, and –CH_3_ analogs were converted to their desired products, particularly at elevated temperatures of 60°C and 80°C. Ligands with *ortho* substituents with Hammett values of + 0.5 or greater gave little to no conversion to the desired PO product. Finally, we found that temperature had a variety of effects with –Cl and –H analogs showing the highest product formation at 60°C; thus, 60°C was used for all reactions to facilitate parallel synthesis. Furthermore, elevated temperatures of 80°C were not universally suitable for all amino acid reactants; optimization of phenylalanine ligand PO2 synthesis showed that temperatures above 60°C gave large quantities of ascididemin side product (Table S1). Using insights from this analysis, we successfully synthesized 12 PO ligands shown in Figure 1B. It is noteworthy that while we achieved high product formation in the crude reaction mixtures, purification proved inefficient, typically providing low percentage yields of the final purified product. These results indicate that future development of PO‐based ligands should prioritize optimization of purification strategies rather than further modification of the condensation conditions.

## 3 DNA Recognition Studies

We next investigated the application of the new PO ligands as Cu‐dependent AMNs. Initially, the ability of the ligands and their corresponding Cu(II) complexes to bind DNA was investigated in situ using a fluorescent intercalator displacement (FID) assay. Here, an intercalating agent that shows enhanced fluorescence when bound to DNA is added to the DNA sample to reach saturation. Addition of a test compound, which competes for DNA binding, then causes ejection of the reporter and the Cheng–Prusoff transformation can be used to calculate an apparent binding constant (*K*_app_) based on the concentration of analyte required to eject 50% of the reporter (*C*_50_) [24]. Typically, ethidium bromide (EtBr) is used as the reporter molecule due to its highly studied DNA binding characteristics and strong fluorescent properties [25]. However, EtBr is mutagenic and we therefore sought to use a safer alternative. Cyanine dyes have long been used as alternatives to EtBr, with commercial dyes like the SYBR series now finding common place in molecular biology staining protocols. Our group recently used thiazole orange (TO) to enhance the DNA binding of triplex forming oligonucleotides and TO has also been used previously in FID assays [26, 27, 28]. Calculation of *K*_app_ requires knowledge of the reporter molecule's binding constant (*K*_b_). Accordingly, we first established the *K*_b_ of TO to calf thymus DNA (ctDNA) using the Bard model [29].

**FIGURE 3:**
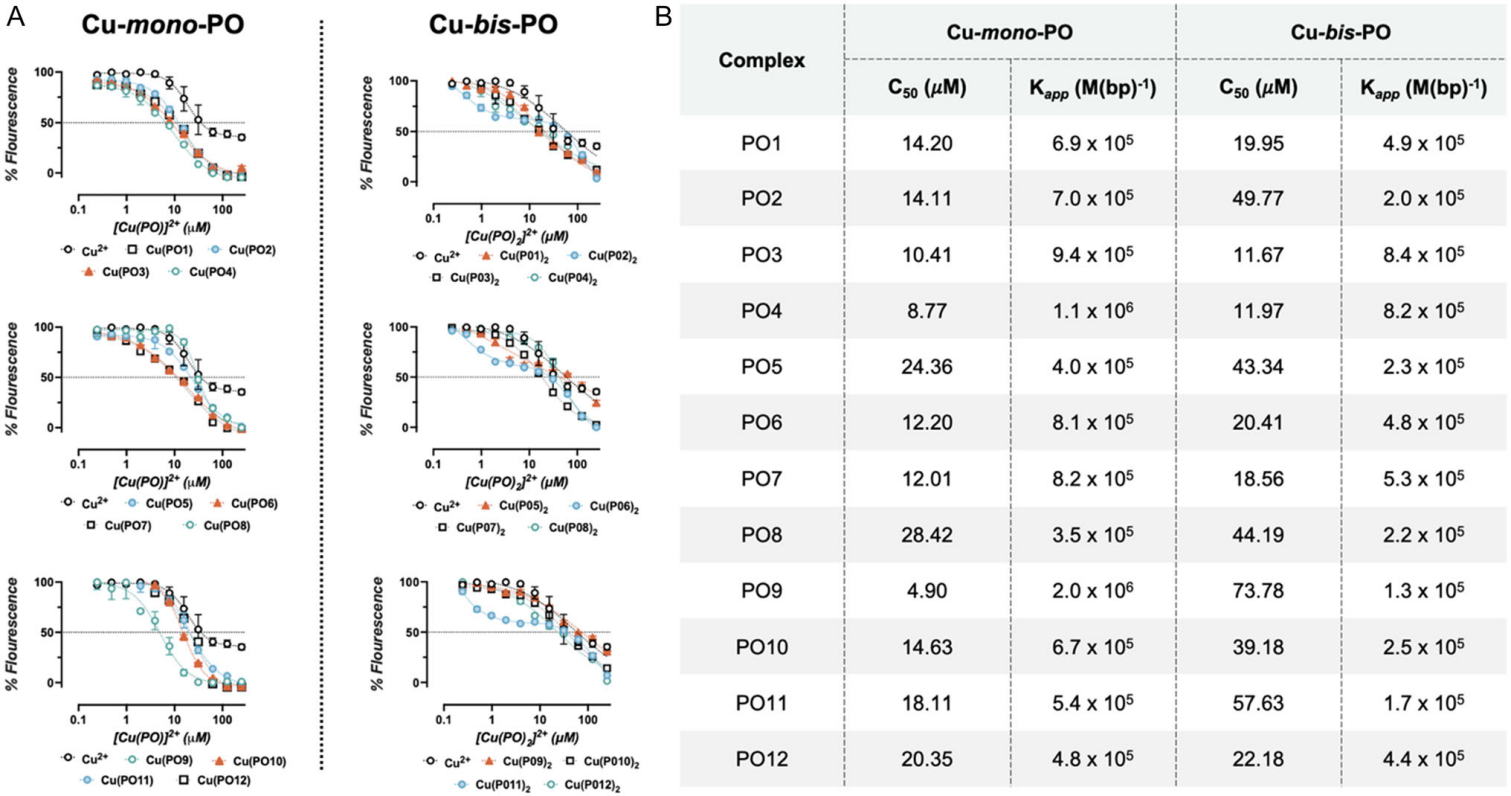
(A) TO displacement assay plots for Cu‐*mono‐*PO and Cu‐*bis‐*PO complexes. Note: Cu^2+^ = Cu(ClO_4_)_2_·6H_2_O. (B) Calculations from the TO displacement assay, showing *C*_50_ and *K*_app_ values for each complex. All error bars indicate ± standard error and all experiments were run in triplicate (*n* = 3).

TO displacement assays were then conducted in a high‐throughput manner to calculate *K*_app_ values of PO ligands and [Cu(PO)_*x*_]^2+^ complexes (where X = 1 or 2), which were prepared in situ. Thus, [Cu(PO)]^2+^ (Cu‐*mono*‐PO) and [Cu(PO)_2_]^2+^ (Cu‐*bis*‐PO) complexes were both examined in this study. Titration of the PO ligands alone resulted in negligible TO displacement and that of Cu^2+^ resulted in only partial quenching. Titration of Cu‐*mono*‐PO complexes, resulted in complete quenching of TO fluorescence. Here, *C*_50_ values were significantly lower than Cu^2+^ alone, with *K*_app_ values ranging from 2.0 × 10^6^ M^−1^ for Cu‐*mono*‐PO9 to 3.5 × 10^5^ M^−1^ for Cu‐*mono*‐PO8 (Figure 3A,B). Cu‐*mono*‐PO9, containing a tryptophan‐PO ligand, showed the tightest binding indicating that the extended aromatic surface provided by the indole moiety is beneficial for binding. Interestingly, complexes of the two aliphatic ligands in the series, PO5 and PO11, were among the lowest *K*_app_ values in the series. Overall, the less polar, more aromatic PO analogs appear to be more efficient DNA binders in the context of 1:1 stoichiometric complexes.

Next, the in situ Cu‐*bis*‐PO complexes, prepared by mixing PO ligands and Cu^2+^ in a 2:1 ratio, were examined. Motivation for testing this ratio stemmed from earlier observations that Cu‐*bis*‐phen complexes generally show enhanced DNA recognition properties [9]. Here, we found that DNA binding was affected by the Cu:PO stoichiometry (Figure 3A,B). Results show that while complexes recording the highest *K*_app_ values were again those incorporating aromatic amino acid derivatives, several complexes demonstrated a decrease in binding affinity relative to their *mono*‐counterparts. It is also noteworthy that the PO9 ligand when complexed 1:1 with Cu^2+^ recorded the highest overall binding affinity, while the 1:2 Cu‐*bis*‐PO9 complex was the weakest binding agent of the *bis‐*PO series. This suggests that while aromatic PO groups enhance DNA recognition, there is an upper limit to their inclusion, beyond which, binding is sterically inhibited.

**FIGURE 4:**
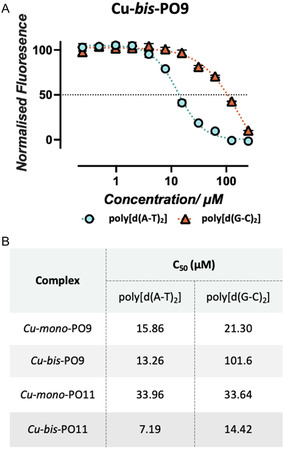
(A) TO fluorescence quenching assay of Cu‐*bis‐*PO9. (B) Numerical results from the TO fluorescence quenching assay, showing the *Q* value for each complex. All error bars indicate ± standard error and all experiements were run in triplicate (*n* = 3).

To investigate the trends in DNA binding further, the tryptophan (PO9) and leucine (PO11) analogs were selected for additional testing to identify the potential binding site interactions. Since [Cu(phen)_2_]^2+^ cleaves DNA through a mechanism of hydrogen atom abstraction from the C1′ position of the deoxyribose ring [10] and is primarily accessible via the minor groove, knowledge of groove binding specificity by new complexes is therefore valuable. Indeed, groove specificity often correlates with sequence specificity, since the accessibility and geometry of the minor and major grooves are determined by the underlying DNA sequence. Thus, duplexes with high A–T content display a deeper and narrower minor groove, with thymine bases presenting a methyl group in the major groove that limits its accessibility. Duplexes with high G–C content lack this methyl group increasing major groove accessibility while presenting a broader, shallower minor groove. We therefore conducted FID assays on *mono*‐ and *bis*‐Cu^2+^ complexes of PO9 and PO11 using synthetic DNA copolymers, poly[d(A‐T)_2_], and poly[d(G‐C)_2_] (Figure 4). Here, both Cu‐*mono*‐PO9 and Cu‐*mono*‐PO11 showed little selectivity with negligible differences in *C*_50_ values for both copolymers, suggesting no groove binding selectivity. However, the *C*_50_ value of Cu‐*bis*‐PO11 for G–C rich DNA was double that for A–T‐rich DNA while Cu‐*bis*‐PO9 returned a *C*_50_ for G–C DNA that was eightfold that for A–T‐rich DNA. Taken together, these data suggest that Cu‐*mono*‐PO complexes show negligible groove specificity while Cu‐*bis*‐PO complexes show significant specificity for the minor groove of A–T‐rich DNA.

## 4 DNA Damage Studies

**FIGURE 5:**
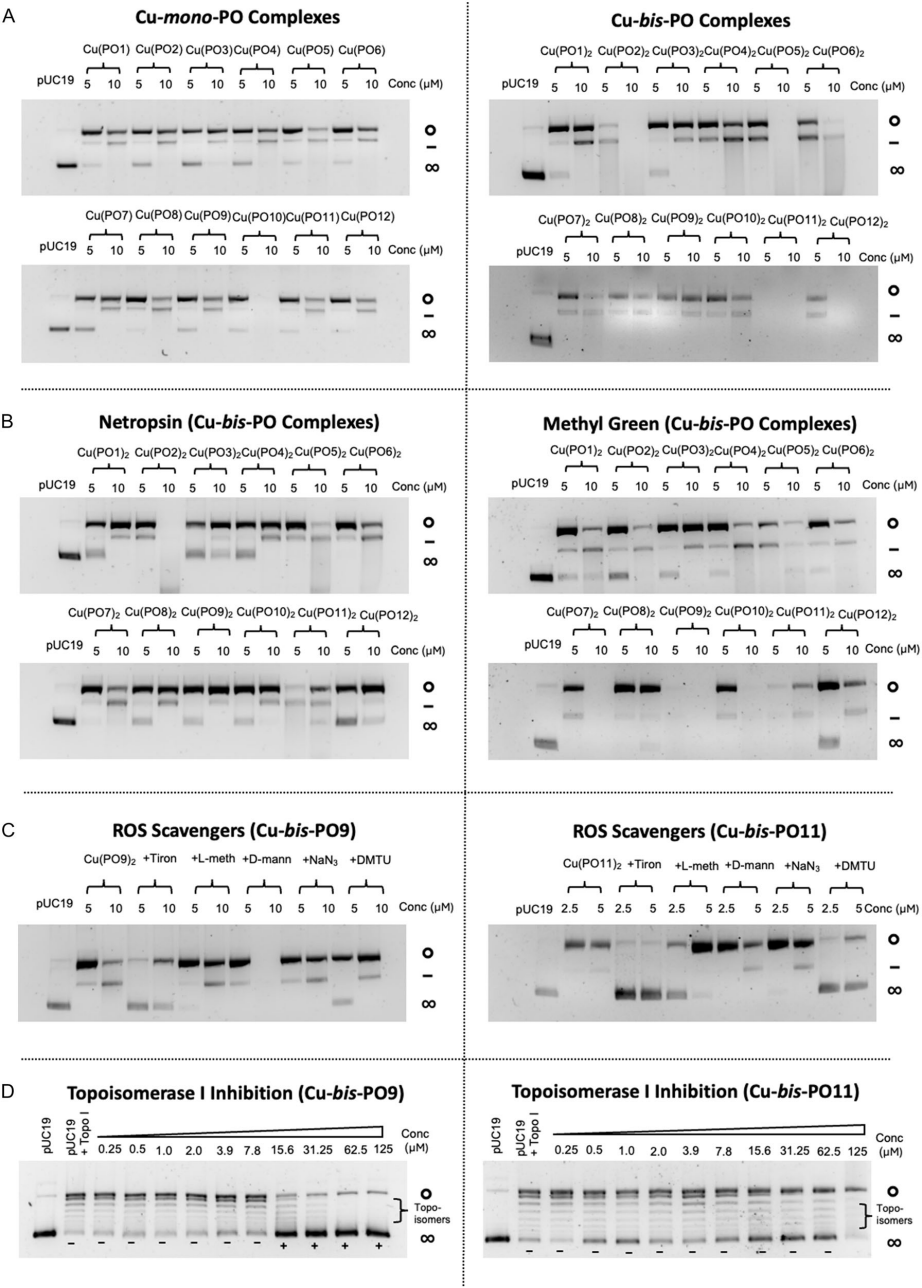
(A) DNA damage visualized by gel electrophoresis of pUC19 exposed to Cu‐*mono‐*PO and Cu‐*bis‐*PO complexes in the presence of added Na‐*L*‐ascorbate (1 mM). (B) DNA damage induced by Cu‐*bis‐*PO9 exposure to pUC19 in the presence of either methyl green or netropsin. (C) DNA damage induced by Cu‐*bis‐*PO9 and Cu‐bis‐PO11 on pUC19 in the presence of specific radical scavengers. (D) Topoisomerase IA inhibition by Cu‐*bis‐*PO9 and Cu‐*bis‐*PO11.

We next examined the DNA cleavage properties of the Cu‐*mono‐*PO and Cu‐*bis‐*PO complexes using gel electrophoresis with supercoiled pUC19. Supercoiled DNA is overwound and therefore has high electrophoretic mobility through the agarose gel. Nicking of a single strand of the DNA duplex causes relaxation to the open circular form that decreases electrophoretic mobility. Cleavage of both strands generates linearized DNA, which has an intermediate mobility, therefore allowing the three forms to be easily separated, visualized, and used as an indication of AMN activity. We first conducted a control experiment with CuCl_2_ in the presence of exogenous reluctant, Na‐*L*‐ascorbate, to provide a basis of comparison for the copper complexes (Figure S28). CuCl_2_ caused significant nicking of the plasmid between 15 and 30 μM and linearization was evident at 250 μM. The Cu‐*mono‐*PO and Cu‐*bis‐*PO complexes were tested at two fixed concentrations of 5 and 10 μM. Here, all complexes were found to have clearly nicked the majority of pUC19 at 5 μM and induced linearization at 10 μM with some variation (Figure 5A). In general, the Cu‐*bis‐*PO complexes were more efficient cleavage agents than the Cu‐*mono‐*PO complexes, in line with the parent phen‐based complexes. Within the Cu‐*bis‐*PO complexes, Cu‐*bis‐*PO11 was the most efficient DNA damaging agent as it was found to completely digest the pUC19 present upon 5 μM exposure. Cu‐*bis‐*PO2, Cu‐*bis‐*PO5 and Cu‐*bis‐*PO12 also displayed potent AMN activity with complete digestion of pUC19 at 10 μM exposure. Cu‐*bis‐*PO1 and Cu‐*bis‐*PO3 were the least efficient AMNs within the series. While the influence of the structure of the PO ligands on the DNA cleavage of the resulting complexes was not immediately obvious, we noted an inverse correlation between DNA cleavage activity and DNA binding affinity for a number of complexes. For example, Cu‐*bis*‐PO2 was among the Cu‐*bis*‐PO complexes with the lowest DNA binding affinity but was among the most efficient cleavage agents. The same is true for Cu‐*bis*‐PO5, Cu‐*bis*‐PO6 and Cu‐*bis*‐PO11.

Next, cleavage experiments for Cu‐*bis*‐PO complexes were examined in the presence of methyl green, a major groove binding agent, and netropsin, a minor groove binding agent (Figure 5B) [30, 31]. In general, cleavage activity of Cu‐*bis*‐PO complexes was inhibited, to varying degrees, in the presence of netropsin and enhanced in the presence of methyl green, with the exceptions of Cu‐*bis*‐PO2, Cu‐*bis*‐PO6 and Cu‐*bis*‐PO8 which showed inhibition by methyl green and enhancement with netropsin. These results indicate that derivatization of the PO ligands significantly impacts groove specific cleavage activity—an observation strongly supported by the early sequence‐specific binding data.

To understand more about the reactive oxygen species (ROS) involved in DNA oxidation pathways of this series, we next examined the cleavage activity Cu‐*bis*‐PO9 and Cu‐*bis*‐PO11 in the presence of ROS scavengers and stabilizers (Figure 5C). Tiron, *L*‐methionine, D‐mannitol, DMTU, and NaN_3_ were selected as they are known to scavenge superoxide, hypochlorous acid, hydroxyl radicals, hydrogen peroxide, and singlet oxygen, respectively [16, 18]. Both complexes were predominantly inhibited by tiron and DMTU which scavenge superoxide and hydrogen peroxide, respectively, implicating the Fenton and Haber–Weiss chemistries in the cleavage mechanism.

Finally, we investigated the ability of Cu‐*bis*‐PO9 and Cu‐*bis*‐PO11 to inhibit DNA unwinding. A useful assay in this regard is the topoisomerase IA (Topo IA) unwinding assay which provides information on both DNA intercalative binding and on nonspecific competition on supercoiled DNA by introducing a transient single‐strand break, allowing controlled rotation of one strand around the other to remove torsional stress before resealing the nick [32]. Through successive rounds of cleavage and religation, Topo IA generates a population of DNA molecules with varying degrees of superhelicity, known as topoisomers, that can be visualized via electrophoresis. Topo IA is specific for negatively supercoiled DNA and intercalators can inhibit Topo IA by unwinding the superhelix, causing a transition from negative to positive supercoiling. In this experiment, we found that Cu‐*bis*‐PO9 was a significantly more efficient inhibitor of Topo IA compared with Cu‐*bis*‐PO11 (Figure 5D). Given their very similar DNA binding affinities, it is unlikely that this difference in Topo IA inhibition is a result of nonspecific competition and suggests that the aromatic tryptophan side chain of Cu‐*bis*‐PO9 is inducing an intercalative binding mode.

## 5 Conclusion

We have optimized the preparation of phenanthroline–oxazine (PO) ligands and found that both the amino acid substrate and temperature are the main influencing parameters, with higher temperatures and negative Hammett values generally yielding higher ratios of product formation. Through this optimization, we synthesized, purified, and characterized 12 PO ligands, nine of which are novel. The synthesis remains relatively challenging from a yield and purification perspective and future efforts in product isolation that avoids isolation via preparative LCMS, such as precipitation and recrysallization, may further refine the preparation and isolation of bioactive PO ligands. Access to PO's that contain more challenging electron withdrawing substituents, such as halo, pseudohalo, or nitro groups, will require alternative methods of preparation, potentially from the aniline PO12 utilizing Sandmeyer‐type chemistry. Our investigation into the DNA binding affinities of the Cu‐*mono‐*PO and Cu‐*bis‐*PO complexes demonstrated that variation in affinity is due to both structural differences within the PO ligands and the number of ligands coordinated to the central metal ion. Cu‐*mono‐*PO9, the tryptophan analog with an extended aromatic system, had the highest DNA binding constant, and our data suggested that it preferentially binds in the minor groove of DNA. Generally, more polar phenylalanine derivatives had lower DNA binding affinities while more lipophilic complexes in the series, such as the leucine or tryptophan derivatives, had higher DNA recognition properties. Interestingly, the binding affinity did not seem to correlate with DNA cleavage. This is evident in complexes with intermediate and low DNA binding constants, such as Cu*‐bis‐*PO2, Cu‐*bis‐*PO5, Cu‐*bis‐*PO11, and Cu‐*bis‐*PO12, which were found to be more active AMNs. Mechanistically, our data suggest that both Cu‐*bis‐*PO9 and Cu‐*bis‐*PO11 cleave DNA using a primarily peroxide‐ and superoxide‐mediated pathway, suggesting a conserved cleavage mechanism within the series consistent with earlier findings by this group [17, 18, 19]. Finally, Topo IA unwinding assays revealed that Cu‐*bis‐*PO9 inhibited Topo IA more efficiently than Cu‐*bis‐*PO11, these data are compatible with an intercalative binding mode for the more aromatic PO derivatives. Overall, these complexes display that structure and activity are clearly linked in terms of DNA binding affinity, and that adaptation is able to enhance the efficiency. In conclusion, these complexes have strong potential in biological application owing to their DNA binding abilities and general AMN activity. As such, they are promising candidates to be hybridized with gene targeting vectors to personalize AMN activity going forward.

## 6 Experimental Section

^1^H and ^13^C NMR spectra were obtained on a Bruker AC 600 MHz NMR spectrometer and processed in MNova (MestreLab). Competitive fluorescence displacement assays were performed using commercial TO (Merck), DNA copolymers poly[d(A–T)_2_] and poly[d(G–C)_2_] (Merck), ctDNA (Fisher Scientific), and 96‐well plates (Merck) were read on a TECAN Spark microplate reader. DNA damage studies were performed using pUC19 (Brennan & Co), topoisomerase IA (Brennan & Co), and methyl green (TCI).

## 7 General Synthesis Method of PO Ligands

1,10‐Phenanthroline‐5,6‐dione (1 eq, 5.15 mmol) was added to a solution of the corresponding amino acid methyl ester (1 eq, 5.15 mmol) and 4‐methylmorpholine (1.1 eq, 5.66 mmol) in DMSO (50 mL) and heated to 60°C for 16 h. The crude product was diluted with DCM, and the organic phase washed × 3 with water (1 × water, 2 × 5% aq. LiCl). After separation, the organic phase was dried over sodium sulfate. The resulting solution was evaporated to dryness *in vacuo* whereby a solid residue remained. The solid was dissolved in DCM and treated with an equal volume of pH 3 citric acid buffer. The organic phase was separated and washed with water. After separation and drying over sodium sulfate, the solvent was removed by evaporation to yield a solid which was further purified by mass directed HPLC.

**PO1**: Formed as a yellow glass (12 mg). ^1^H NMR (600 MHz, CDCl_3_) δ 9.16 (dd, *J* = 4.3, 1.8 Hz, 1H), 9.09 (dd, *J* = 4.3, 1.7 Hz, 1H), 9.01 (dd, *J* = 8.2, 1.7 Hz, 1H), 8.60 (dd, *J* = 8.3, 1.8 Hz, 1H), 7.69 (dd, *J* = 8.3, 4.3 Hz, 1H), 7.61 (dd, *J* = 8.2, 4.3 Hz, 1H), 7.30–7.27 (m, 2H), 6.77–6.74 (m, 2H), 6.57 (s, 1H), 3.98 (s, 3H), 3.69 (d, *J* = 6.0 Hz, 3H). ^13^C NMR (151 MHz, CDCl_3_) δ 163.49, 160.84, 151.79, 149.16, 148.94, 147.47, 143.09, 139.30, 131.55, 130.82, 128.94, 126.79, 126.73, 123.89, 123.27, 122.66, 121.89, 114.58, 72.44, 55.34, 53.39. UPLC–MS: [M+H]^+^ m/z found: 400.2, Calc: 400.1 (96% purity).

**PO2**: Formed as a brown glass (16 mg). ^1^H NMR (600 MHz, CDCl_3_) δ 9.17 (dd, *J* = 4.3, 1.8 Hz, 1H), 9.08 (dd, *J* = 4.3, 1.8 Hz, 1H), 8.98 (dd, *J* = 8.2, 1.7 Hz, 1H), 8.64 (dd, *J* = 8.2, 1.8 Hz, 1H), 7.68 (dd, *J* = 8.2, 4.3 Hz, 1H), 7.63 (dd, *J* = 8.2, 4.3 Hz, 1H), 7.38–7.34 (m, 2H), 7.26 (s, 3H), 6.65 (s, 1H), 4.00 (s, 3H). ^13^C NMR (151 MHz, CDCl_3_) δ 163.42, 151.75, 149.10, 148.64, 147.36, 142.99, 139.21, 134.86, 131.41, 130.76, 129.72, 129.06, 126.98, 126.57, 123.80, 123.23, 122.60, 121.62, 72.42, 67.09. UPLC–MS: [M+H]^+^ m/z found: 370.1 Calc: 370.1 (99% purity).

**PO3**: Formed as a yellow solid (11 mg). ^1^H NMR (600 MHz, CDCl_3_) δ 9.16 (dd, *J* = 4.3, 1.8 Hz, 1H), 9.12 (dd, *J* = 4.3, 1.7 Hz, 1H), 9.08 (dd, *J* = 8.2, 1.7 Hz, 1H), 8.53 (dd, *J* = 8.3, 1.8 Hz, 1H), 7.74 (dd, *J* = 8.2, 4.3 Hz, 1H), 7.61 (dd, *J* = 8.2, 4.3 Hz, 1H), 7.12–7.09 (m, 1H), 6.92 (d, *J* = 7.9 Hz, 1H), 6.85 (s, 1H), 6.78 (dt, *J* = 7.9, 1.3 Hz, 1H), 3.99 (s, 3H), 2.75 (s, 3H), 2.22 (s, 3H).^13^C NMR (151 MHz, CDCl_3_) δ 163.24, 151.54, 149.00, 142.95, 140.17, 137.62, 132.58, 132.56, 132.48, 132.30, 131.34, 131.32, 130.66, 127.55, 126.87, 126.85, 125.83, 126.64, 123.76, 123.02, 70.00, 53.25, 21.08, 19.33. UPLC–MS: [M+H]^+^ m/z found: 398.2 Calc: 398.3 (97% purity).

**PO4**: Formed as a yellow solid (4 mg). ^1^H NMR (600 MHz, CDCl_3_) δ 9.12 (dd, *J* = 4.3, 1.8 Hz, 1H), 9.03 (dd, *J* = 4.3, 1.8 Hz, 1H), 8.90 (dd, *J* = 8.2, 1.7 Hz, 1H), 8.55 (dd, *J* = 8.2, 1.8 Hz, 1H), 7.63 (dd, *J* = 8.2, 4.3 Hz, 1H), 7.58 (dd, *J* = 8.2, 4.3 Hz, 1H), 7.24–7.23 (m, 1H), 7.23–7.21 (m, 1H), 7.16 (d, *J* = 2.1 Hz, 1H), 7.15 (d, *J* = 2.0 Hz, 1H), 6.54 (s, 1H), 3.94 (s, 3H). ^13^C NMR (151 MHz, CDCl_3_) δ 163.32, 151.92, 149.27, 148.14, 143.08, 133.37, 131.30, 130.73, 129.35, 128.40, 126.47, 123.86, 123.66, 123.42, 123.29, 122.55, 122.30, 121.48, 71.74, 53.39. UPLC–MS: [M+H]^+^ m/z found: 404.1 Calc: 404.2 (95% purity).

**PO5**: Formed as a brown glass (9 mg). ^1^H NMR (600 MHz, CDCl_3_) δ 9.20 (dd, *J* = 4.3, 1.8 Hz, 1H), 9.08 (dd, *J* = 4.3, 1.7 Hz, 1H), 8.89 (dd, *J* = 8.2, 1.7 Hz, 1H), 8.62 (dd, *J* = 8.2, 1.8 Hz, 1H), 7.66 (dd, *J* = 8.2, 4.3 Hz, 2H), 3.99 (s, 3H), 1.78 (s, 6H). ^13^C NMR (151 MHz, CDCl_3_) δ 163.22, 154.22, 151.49, 148.82, 147.22, 142.82, 139.47, 131.12, 130.56, 126.53, 123.66, 123.02, 121.87, 121.60, 76.17, 52.84, 24.27. UPLC–MS: [M+H]^+^ m/z found: 322.1 Calc: 322.1 (96% purity).

**PO6**: Formed as a green solid (5 mg). ^1^H NMR (600 MHz, CDCl_3_) δ 9.12 (dd, *J* = 4.3, 1.8 Hz, 1H), 9.02 (dd, *J* = 4.3, 1.7 Hz, 1H), 8.91 (dd, *J* = 8.3, 1.8 Hz, 1H), 8.59 (dd, *J* = 8.2, 1.8 Hz, 1H), 7.60 (ddd, *J* = 21.7, 8.2, 4.3 Hz, 2H), 7.09 (t, *J* = 8.2 Hz, 1H), 6.88–6.81 (m, 2H), 6.73 (ddd, *J* = 8.4, 2.5, 1.1 Hz, 1H), 6.56 (s, 1H), 3.94 (s, 3H), 3.60 (s, 3H). ^13^C NMR (151 MHz, CDCl_3_) δ 163.44, 159.91, 151.75, 149.12, 148.55, 147.38, 143.01, 139.21, 136.30, 131.38, 130.77, 130.13, 126.57, 123.79, 123.22, 122.66, 121.60, 119.06, 114.60, 113.14, 72.23, 55.19, 53.33. UPLC–MS: [M+H]^+^ m/z found: 400.1 Calc: 400.2 (97% purity).

**PO7**: Formed as an orange solid (23 mg). ^1^H NMR (600 MHz, CDCl_3_) δ 9.22 (dd, *J* = 4.3, 1.8 Hz, 1H), 9.14 (dd, *J* = 4.3, 1.7 Hz, 1H), 9.02 (dd, *J* = 8.2, 1.7 Hz, 1H), 8.65 (dd, *J* = 8.2, 1.8 Hz, 1H), 7.71 (ddd, *J* = 32.0, 8.2, 4.3 Hz, 2H), 7.41–7.34 (m, 2H), 7.00–6.94 (m, 2H), 6.65 (s, 1H), 4.03 (s, 3H). ^13^C NMR (151 MHz, CDCl_3_) δ 163.32, 151.85, 149.21, 148.39, 131.33, 130.75, 129.33, 129.20, 129.13, 129.08, 126.51, 123.85, 123.80, 123.50, 123.27, 122.52, 121.56, 121.30, 116.27, 116.13, 71.80, 53.36. UPLC–MS: [M+H]^+^ m/z found: 388.1 Calc: 388.2 (96% purity).

**PO8**: Formed as an orange solid (25 mg). ^1^H NMR (600 MHz, CDCl_3_) δ 8.76 (dd, *J* = 8.2, 1.7 Hz, 1H), 8.48–8.43 (m, 2H), 8.34–8.28 (m, 1H), 7.38 (dd, *J* = 8.2, 4.3 Hz, 1H), 7.30 (d, *J* = 2.1 Hz, 1H), 7.16 (dd, *J* = 8.2, 4.3 Hz, 1H), 6.99–6.92 (m, 2H), 6.59 (s, 1H), 4.04 (s, 3H). ^13^C NMR (151 MHz, DMSO) δ 163.16, 150.29, 149.11, 147.46, 146.88, 145.99, 139.04, 131.44, 125.81, 124.62, 124.26, 122.12, 121.98, 121.80, 121.71, 119.33, 116.34, 114.97, 72.71, 53.42, 40.53, 31.16. UPLC–MS: [M+H]^+^ m/z found: 402.1 Calc: 402.2 (95% purity).

**PO9**: Formed as an orange solid (135 mg). ^1^H NMR (600 MHz, CDCl_3_) δ 9.13 (ddd, *J* = 4.5, 2.9, 1.7 Hz, 2H), 9.09 (dd, *J* = 8.2, 1.7 Hz, 1H), 8.58 (dd, *J* = 8.2, 1.8 Hz, 1H), 8.11 (s, 1H), 8.00–7.95 (m, 1H), 7.74 (dd, *J* = 8.2, 4.3 Hz, 1H), 7.57 (dd, *J* = 8.3, 4.3 Hz, 1H), 7.35–7.31 (m, 1H), 7.27–7.22 (m, 2H), 7.10 (d, *J* = 0.7 Hz, 1H), 7.06 (dd, *J* = 2.8, 0.7 Hz, 1H), 4.00 (s, 3H). ^13^C NMR (151 MHz, DMSO) δ 163.16, 150.29, 149.11, 147.46, 146.88, 145.99, 139.04, 131.44, 130.38, 127.00, 125.81, 124.62, 124.26, 122.12, 121.98, 121.80, 121.71, 119.33, 66.99, 53.39, 49.06, 40.91, 40.54, 31.15. UPLC–MS: [M+H]^+^ m/z found: 409.1 Calc: 409.2 (95% purity).

**PO10**: Formed as a yellow solid (7 mg). ^1^H NMR (600 MHz, CDCl_3_) δ 9.10 (dd, *J* = 4.3, 1.8 Hz, 1H), 9.02 (dd, *J* = 4.3, 1.7 Hz, 1H), 8.97–8.91 (m, 1H), 8.54 (dd, *J* = 8.3, 1.8 Hz, 1H), 7.63 (dd, *J* = 8.2, 4.3 Hz, 1H), 7.56 (dd, *J* = 8.2, 4.3 Hz, 1H), 7.18–7.15 (m, 2H), 6.66–6.62 (m, 2H), 6.50 (s, 1H), 3.92 (s, 3H). ^13^C NMR (151 MHz, CDCl_3_) δ 151.67, 149.02, 147.46, 146.88, 145.99, 139.04, 131.48, 131.44, 130.74, 129.03, 125.81, 124.62, 123.79, 123.17, 122.12, 121.98, 121.80, 116.01, 72.29, 53.28. UPLC–MS: [M+H]^+^ m/z found: 386.1 Calc: 386.2 (99% purity).

**PO11**: Formed as a brown glass (34 mg). ^1^H NMR (600 MHz, CDCl_3_) δ 9.25 (ddd, *J* = 4.4, 1.8, 0.7 Hz, 1H), 9.13 (ddd, *J* = 4.3, 1.8, 0.7 Hz, 1H), 8.96 (ddd, *J* = 8.2, 1.8, 0.7 Hz, 1H), 8.71 (ddd, *J* = 8.2, 1.8, 0.7 Hz, 1H), 7.72 (dddd, *J* = 8.3, 5.0, 4.3, 0.7 Hz, 2H), 5.38 (dd, *J* = 8.4, 0.7 Hz, 1H), 4.04 (d, *J* = 0.7 Hz, 3H), 1.14 (s, 1H), 1.07–0.99 (m, 6H). ^13^C NMR (151 MHz, CDCl_3_) δ 167.26, 163.97, 151.55, 149.35, 148.86, 144.31, 139.25, 131.21, 130.67, 128.54, 123.51, 122.89, 121.51, 76.28, 53.20, 29.36, 27.88, 22.47, 17.79. UPLC–MS: [M+H]^+^ m/z found: 336.1 Calc: 336.2 (96% purity).

**PO12**: Formed as an orange solid (21 mg). ^1^H NMR (600 MHz, CDCl_3_) δ 9.17 (s, 1H), 9.10 (d, *J* = 3.9 Hz, 1H), 9.02 (d, *J* = 8.2 Hz, 1H), 8.60 (d, *J* = 8.2 Hz, 1H), 7.70 (dd, *J* = 8.3, 4.3 Hz, 1H), 7.61 (dd, *J* = 8.0, 4.2 Hz, 1H), 7.15–7.11 (m, 2H), 6.56–6.47 (m, 3H), 3.97 (s, 3H). ^13^C NMR (151 MHz, CDCl_3_) δ 163.41, 151.57, 148.99, 148.93, 147.99, 147.33, 142.96, 131.46, 130.80, 130.67, 128.94, 126.70, 124.10, 123.69, 123.06, 122.52, 121.92, 115.06, 72.74, 53.17. UPLC–MS: [M+H]^+^ m/z found: 385.1 Calc: 385.2 (98% purity).

## 8 DNA Binding Assays

A 10 mg/mL solution of TO was prepared in DMSO diluted 1000x in water and quantified using absorbance, *ε* = 63,000 M^−1^ cm^−1^ at 500 nm [33].

### 8.1 Continuous Variation Analysis

Two equimolar stock solutions of ctDNA and TO were prepared in 80 mM HEPES containing 25 mM NaCl, at pH 7.4. Different volumes of each stock solution were then combined in a 96‐well transparent microplate to give a total volume of 100 µL per well, maintaining a constant total concentration of 15 µM.The plate was allowed to equilibrate at room temperature for 30 min before the fluorescence at 540 nm was recorded after excitation at 475 nm. Values from TO‐bound ctDNA experiments were compared to values from a control prepared plate, prepared under identical conditions, but without ctDNA. The difference in fluorescence was plotted against the mole fraction of TO in each well. A linear least‐squares fitting routine was employed in GraphPad Prism to determine the inflection point (Figure S25). The ratio of the inflection point was then used to identify the binding stoichiometry.

### 8.2 Thiazole Orange and ctDNA Equilibrium Binding Titration

A triplicate serial dilution of ctDNA was prepared in a 96‐well plate in 80 mM HEPES with 25 mM NaCl, at pH 7.4 to a final volume of 50 μL. Then, 50 μL of a 500 nM TO solution was added to give a final concentration of 250 nM TO. Blank wells contained no ctDNA substrate. Fluorescence at 540 nm was recorded after excitation at 475 nm and used to calculate the fraction of TO bound using Equation (1) [34].

(1) $$\frac{\left(\frac{F_{\text{sample}}}{F_{\text{blank}}}\right)}{\left(\frac{F_{\max}}{F_{\text{blank}}}\right)}=\text{Fraction Bound}$$

where *F*_sample_ is the fluorescence in the sample well, *F*_blank_ is the fluorescence of a blank well (no ctDNA substrate), and *F*_max_ is the average value of those wells that contained the highest ctDNA concentration. The Bard model (Equations (2) and (3) was applied to the binding curve using a binding site size of 12 bp (found from continuous variation) to calculate the *K*_b_ of TO bound to ctDNA.

(2) $$Y=\frac{\left(b-\sqrt{b^{2}-2K^{2}DN^{-1}}\right)}{2KD}$$

(3) $$b=1+KD+\frac{KX}{2N}$$

where *Y* is the fraction bound to ctDNA, *K* is the binding affinity, *D* is the concentration of TO (M), *N* is the binding site size, and *X* is the concentration of DNA (M).

### 8.3 Fluorescent Intercalator Displacement Using Thiazole Orange

In 96‐well plates, serial dilutions of PO ligands, Cu(ClO_4_)_2_·6H_2_O and [Cu(PO)_*x*_]^2+^ complexes (where *x* = 1 or 2) were prepared in a volume of 50 μL using 80 mM HEPES, 20 mM NaCl, at pH 7.4 with 5% DMSO. To this mixture, 50 μL of a working solution containing 25.2 μM TO and 20 μM ctDNA in the same buffer was added to give final conditions of 12.6 μM TO, 10 μM ctDNA together with varying analyte concentration in a total volume of 100 μL. Blank wells were prepared to contain no ctDNA and control wells contained no titrant or analyte. Plates were allowed to equilibrate for 30 min prior to fluorescence measurements using excitation at 475 nm and detection 540 nm. The relative fluorescence percentage was calculated using Equation (4) where *Fs* is the fluorescence of the sample in question, *Fb* is the average value of blank samples, and *Fc* is the value of control samples (blank and control samples included in each individual plate).

(4) $$\%F=\frac{\left(Fs-Fb\right)}{\left(Fc-Fb\right)}\times 100$$

Each sample was measured in triplicate and the apparent binding constants were calculated using Equation (5) where *K*_b_ is the binding constant of TO (7.8 × 10^5^ M(bp)^−1^), [TO] is the concentration of TO (12.6 μM), and *C*_50_ is the concentration of analyte required to reduce fluorescence by 50%.

(5) $$K_{\mathrm{app}}=K_{b}\times \frac{\left[\mathrm{TO}\right]}{C_{50}}$$

Fluorescence quenching of poly[d(A–T)_2_] and poly[d(G–C)_2_] was conducted in an equivalent manner but with a final TO concentration of 2.52 μM and 2.00 μM of poly[d(A–T)_2_] and poly[d(G–C)_2_].

### 8.4 DNA Cleavage by Agarose Gel Electrophoresis

Cleavage reactions were performed in 100 µL Eppendorf tubes using a final volume of 20 µL which contained 400 ng of supercoiled pUC19 DNA, 1 mM Na‐*L*‐ascorbate, 25 mM NaCl in 80 mM HEPES buffer (pH = 7.4) and 5.0 or 10 µM of the Cu–PO complex prepared in situ. After the addition of pUC19, the samples were incubated for 30 min at 37°C in a ThermoMixer, quenched with 6x DNA loading dye (Thermo Fisher R0611) and loaded onto a 1.3% agarose gel, prepared using 1x TAE buffer. The gel was then run at 70 V for 90 min. Cleavage reactions with ROS scavengers were prepared to contain 10 mM of the ROS scavenger by addition of 1 μL of a 200 mM stock solution prior to DNA addition. Reactions probing the cleavage site were examined by preparing reaction mixtures to contain 16 μM and 8 μM of methyl green and netropsin, respectively, from stock solutions prepared in 80 mM HEPES buffer (pH = 7.4).

### 8.5 Topoisomerase IA Inhibition

A topoisomerase IA inhibition assay was carried out as previously described with modification [19]. Experiments were carried out by adding 400 ng of pUC19 DNA to sample tubes containing varying concentrations of test compound, prepared by serial dilution in 80 mM HEPES (25 mM NaCl, pH = 7.4) with 1× CutSmart in a final volume of 20 μL. Reactions were incubated in darkness for 30 min at room temperature. Next, the topoisomerase IA enzyme (1 unit) was added to all samples with exception of the control, and the mixtures were incubated for 20 min at 37°C. Topo IA was deactivated by heating to 65°C for 25 min. Next, 4 μL of 6× Fermentas loading buffer containing 10 mM Tris‐HCl, 0.03% bromophenol blue, 0.03% xylene cyanole FF, 60% glycerol, and 60 mM EDTA was added and the samples were then loaded onto 1.3% native agarose gel in 1× Tris‐borate‐EDTA (TBE) buffer. Electrophoresis was performed at 60 V for 2.5 h in 1× TBE buffer. The gel was then visualized by staining with SYBR safe (20 μL of dye in 100 mL H_2_O) for 30 min, followed by soaking in a water bath for 15 min, and then imaged using a UV transilluminator (G:Box mini 9, GeneSys software, Syngene).

## Supporting Information

Additional supporting information (SI) can be found online in the Supporting Information section.

## Conflicts of Interest

The authors declare no conflicts of interest.

## Supporting information

Supplementary Material

## References

[1] A. Bencini and V. Lippolis , “1,10‐Phenanthroline: A Versatile Building Block for the Construction of Ligands for Various Purposes,” Coordination Chemistry Reviews 254 (2010): 2096–2180.

[2] P. G. Sammes and G. Yahioglu , “1,10‐Phenanthroline: A Versatile Ligand,” Chemical Society Reviews 23 (1994): 327–334.

[3] Z. Molphy , A. Prisecaru , C. Slator , et al., “Copper Phenanthrene Oxidative Chemical Nucleases,” Inorganic Chemistry 53 (2014): 5392–5404. 24806421 10.1021/ic500914j

[4] A. E. Friedman , J. C. Chambron , J.‐P. Sauvage , N. J. Turro , and J. K. Barton , “A Molecular Light Switch for DNA: Ru(bpy)_2_(dppz)^2+^ ,” Journal of the American Chemical Society, 112 (1990): 4960–4962.

[5] C. Queffelec , P. B. Pati , and Y. Pellegrin , “Fifty Shades of Phenanthroline: Synthesis Strategies to Functionalize 1,10‐Phenanthroline in All Positions,” Chemical Reviews 124 (2024): 6700–6902. 38747613 10.1021/acs.chemrev.3c00543

[6] A. Gibney , and A. Kellett , “Gene Editing with Artificial DNA Scissors,” Chemistry A European Journal 30 (2024): e202401621. 38984588 10.1002/chem.202401621

[7] V. D’Aurora , A. M. Stern , and D. S. Sigman , “Inhibition of DNA Polymerase I by 1,10‐Phenanthroline,” Biochemical and Biophysical Research Communications 78 (1977): 170–176. 334165 10.1016/0006-291x(77)91236-0

[8] V. D’Aurora , A. M. Stern , and D. S. Sigman , “1,10‐Phenanthroline‐Cuprous Ion Complex, A Potent Inhibitor of DNA and RNA‐Polymerases,” Biochemical and Biophysical Research Communications 80 (1978): 1025–1032. 346019 10.1016/0006-291x(78)91348-7

[9] D. S. Sigman , D. R. Graham , V. D’Aurora , and A. M. Stern , “Oxygen‐Dependent Cleavage of DNA by the 1,10‐Phenanthroline cuprous Complex. Inhibition of *Escherichia Coli* DNA Polymerase I.,” Journal of Biological Chemistry 254 (1979): 12269–12272. 387784

[10] D. S. Sigman , “Nuclease Activity of 1,10‐Phenanthroline Copper‐Ion,” Accounts of Chemical Research 19 (1986): 180–186.

[11] T. J. P. McGivern , S. Afsharpour , and C. J. Marmion , “Copper Complexes as Artificial Metallonucleases: From Sigman's Reagent to Next Generation Anti‐Cancer Agent?,” Inorganic Chimica Acta 472 (2018): 12–39.

[12] Í.P. de Souza , B. d. P. Machado , A. B. de Carvalho , et al., “Exploring the DNA Binding, Oxidative Cleavage, and Cytotoxic Properties of New Ternary Copper(II) Compounds Containing 4‐Aminoantipyrine and N,N‐Heterocyclic Co‐Ligands,” Journal of Molecular Structure 1178 (2019): 18–28.

[13] N. Z. Fantoni , Z. Molphy , S. O’Carroll , et al., “Polypyridyl‐Based Copper Phenanthrene Complexes: Combining Stability with Enhanced DNA Recognition,” Chemistry. A European Journal 27 (2021): 971–983. 32519773 10.1002/chem.202001996

[14] Z. Molphy , D. Montagner , S. S. Bhat , et al., “A Phosphate‐Targeted Dinuclear Cu(II) Complex Combining Major Groove Binding and Oxidative DNA Cleavage,” Nucleic Acids Research 46 (2018): 9918–9931. 30239938 10.1093/nar/gky806 PMC6212767

[15] Z. Molphy , C. Slator , C. Chatgilialoglu , and A. Kellett , “DNA Oxidation Profiles of Copper Phenanthrene Chemical Nucleases,” Frontiers in Chemistry 3 (2015): 28. 25954741 10.3389/fchem.2015.00028 PMC4404973

[16] C. Slator , Z. Molphy , V. McKee , C. Long , T. Brown , and A. Kellett , “Di‐Copper Metallodrugs Promote NCI‐60 Chemotherapy via Singlet Oxygen and Superoxide Production with Tandem TA/TA and AT/AT Oligonucleotide Discrimination,” Nucleic Acids Research 46 (2018): 2733–2750. 29474633 10.1093/nar/gky105 PMC5888725

[17] A. Gibney , R. E. F. de Paiva , V. Singh , et al., “A Click Chemistry‐Based Artificial Metallo‐Nuclease,” Angewandte Chemie International Edition 62 (2023): e202305759. 37338105 10.1002/anie.202305759

[18] N. McStay , C. Slator , V. Singh , A. Gibney , F. Westerlund , and A. Kellett , “Click and Cut: A Click Chemistry Approach to Developing Oxidative DNA Damaging Agents,” Nucleic Acids Research 49 (2021): 10289–10308. 34570227 10.1093/nar/gkab817 PMC8501983

[19] S. Poole , O. A. Aning , V. McKee , et al., “Design and In Vitro Anticancer Assessment of a Click Chemistry‐Derived Dinuclear Copper Metallo‐Nuclease,” Nucleic Acids Research 53 (2025): gkae1250. 39777469 10.1093/nar/gkae1250 PMC11705080

[20] A. Alcalde‐Ordóñez , N. Barreiro‐Piñeiro , B. McGorman , et al., “A Copper(II) Peptide Helicate Selectively Cleaves DNA Replication Foci in Mammalian Cells,” Chemical Science 14 (2023): 14082–14091, 10.1039/d3sc03303a. 38098723 PMC10718067

[21] A. Gibney , M. Sidarta , E. Delahunt , et al., “Expanding the DNA Damaging Potential of Artificial Metallo‐Nucleases with Click Chemistry,” Nature Communications 17 (2026): 2309, 10.1038/s41467-026-68911-5. PMC12976370 41634027

[22] M. McCann , J. McGinley , K. Ni , et al., “A New Phenanthroline‐Oxazine Ligand: Synthesis, Coordination Chemistry and Atypical DNA Binding Interaction,” Chemical Communications 49 (2013): 2341–2343. 23407675 10.1039/c3cc38710k

[23] M. Ahmed , D. Rooney , M. McCann , J. Casey , K. O’Shea , and B. Twamley , “Tuning the Reaction Pathways of Phenanthroline‐Schiff Bases: Routes to Novel Phenanthroline Ligands,” Dalton Transactions 48 (2019): 15283–15289. 31580366 10.1039/c9dt03084k

[24] Y. Cheng and W. H. Prusoff , “Relationship between Inhibition Constant (K_i_) and Concentration of Inhibitor which causes 50 per cent Inhibition (I_50_) of an Enzymatic‐Reaction,” Biochemical Pharmacology 22 (1973): 3099–3108. 4202581 10.1016/0006-2952(73)90196-2

[25] A. Kellett , Z. Molphy , C. Slator , V. McKee , and N. P. Farrell , “Molecular Methods for Assessment of Non‐Covalent Metallodrug–DNA Interactions,” Chemical Society Reviews 48 (2019): 971–988. 30714595 10.1039/c8cs00157j PMC6657641

[26] D. L. Boger and W. C. Tse , “Thiazole Orange as the Fluorescent Intercalator in a High Resolution FID Assay for Determining DNA Binding Affinity and Sequence Selectivity of Small Molecules,” Bioorganic & Medicinal Chemistry 9 (2001): 2511–2518. 11553493 10.1016/s0968-0896(01)00243-7

[27] J. Hennessy , P. Klimkowski , D. Singleton , et al., “Thiazole Orange‐Carboplatin Triplex‐Forming Oligonucleotide (TFO) Combination Probes Enhance Targeted DNA Crosslinking,” RSC Medicinal Chemistry 15 (2024): 485–491. 38389892 10.1039/d3md00548h PMC10880910

[28] N. Z. Fantoni , B. McGorman , Z. Molphy , et al., “Development of Gene‐Targeted Polypyridyl Triplex‐Forming Oligonucleotide Hybrids,” ChemBioChem 21 (2020): 3563–3574. 32755000 10.1002/cbic.202000408

[29] M. T. Carter , M. Rodriguez , and A. J. Bard , “Voltammetric Studies of the Interaction of Metal Chelates with DNA. 2. Tris‐Chelated Complexes of Cobalt(III) and Iron(II) with 1,10‐Phenanthroline and 2,2′‐Bipyridine,” Journal of the American Chemical Society 111 (1989): 8901–8911.

[30] S. K. Kim and B. Nordén , “Methyl Green. A DNA Major‐Groove Binding‐Drug,” FEBS Letters 315 (1993): 61–64. 8416812 10.1016/0014-5793(93)81133-k

[31] M. Poot , K. Kausch , J. Köhler , T. Haaf , and H. Hoehn , “The Minor‐Groove Binding Ligands Netropsin, Distamycin‐A and Berenil Case Polyploidization via Impairment of the G2 Phase of the Cell‐Cycle,” Cell Structure and Function 15 (1990): 151–157. 1697788 10.1247/csf.15.151

[32] M. G. Krokidis , Z. Molphy , E. K. Efthimiadou , et al., “Assessment of DNA Topoisomerase I Unwinding Activity, Radical Scavenging Capacity, and Inhibition of Breast Cancer Cell Viability of N‐Alkyl‐Acridones and N,N‐Dialkyl‐9,9‐Biacridylidenes,” Biomolecules 9 (2019): 177. 31072044 10.3390/biom9050177 PMC6572364

[33] S. Das and P. Purkayastha , “Modulating Thiazole Orange Aggregation in Giant Lipid Vesicles: Photophysical Study Associated with FLIM and FCS,” ACS Omega 2 (2017): 5036–5043. 31457780 10.1021/acsomega.7b00899 PMC6641685

[34] K. R. Fox , Drug–DNA Interaction Protocols (Totowa, NJ: Humana Press, 1997).

