# Supplementary material for: Accessing New Phenanthroline–Oxazine Scaffolds as Copper‐Dependent DNA Damaging Probes
Source: Chembiochem. 2026 Apr 17;27(8):e202500874. doi: 10.1002/cbic.202500874 (PMC13089940; doi:10.1002/cbic.202500874)
Supplement: Supplementary file 1 — Supplementary Material [file CBIC-27-e202500874-s001.pdf]

Supplementary Information for:

Accessing New Phenanthroline-Oxazine Scaffolds as Copper-Dependent DNA Damaging Probes

Rebecca Lynn [a], Alex Gibney[a], Eva Delahunt[a], Stephen MacDonald-Brown[b], Carlos Lence[b], Miles Kenny[b], Matt Allen[b], Ravil Khaybullin[b], Iva Lukac[b], Andrew Jordan[b] and Andrew Kellett*[a]

[a] School of Chemical Sciences, Life Sciences Institute and SFI Solid State Pharmaceutical Centre (SSPC), Dublin City University, Glasnevin, Dublin 9, Ireland.
E-mail: andrew.kellett@dcu.ie

[b] Charnwood Discovery, Charnwood Campus, 9 Summerpool Road, Loughborough, LE11 5RD.

Table of Contents

S-1 Synthetic Condition Screening 3

S-2: ^{1}H and ^{13}C NMR Spectra 4

S-3: TO Binding Constant Evaluation with CT DNA 16

S-4: Gel Electrophoresis 17

S-1 Synthetic Condition Screening

Table S1: Results of condition screening for reaction of tyrosine methyl ester with phendione. Product is the corresponding PO ligand and byproduct is the corresponding ascididemin analogue. Percentages determined by UPLC-MS.

Run	Temp °C	Base Eq	Amino Acid Eq	%Product	%Byproduct
1	90	2	2	85.31	4.85
2	90	3	1	76.23	4.04
3	90	2	1	76.37	3.79
4	55	2.5	1.5	58.66	0
5	20	2	2	2.69	0
6	55	2.5	1.5	58.51	0
7	20	3	2	2.08	0
8	20	3	1	0.99	0
9	20	2	1	1.18	0
10	90	2	1	79.61	4.36
11	20	3	2	2.57	0
12	90	2	2	80.72	6.26
13	90	3	2	74.73	5.86
14	20	2	1	1.17	0
15	55	2.5	1.5	57.01	0
16	20	3	1	1.01	0
17	90	3	2	75.58	5.83
18	20	2	2	2.43	0
19	90	3	1	73.5	3.97
20	55	2.5	1.5	59.17	0

S-2: ^{1}H and ^{13}C NMR Spectra

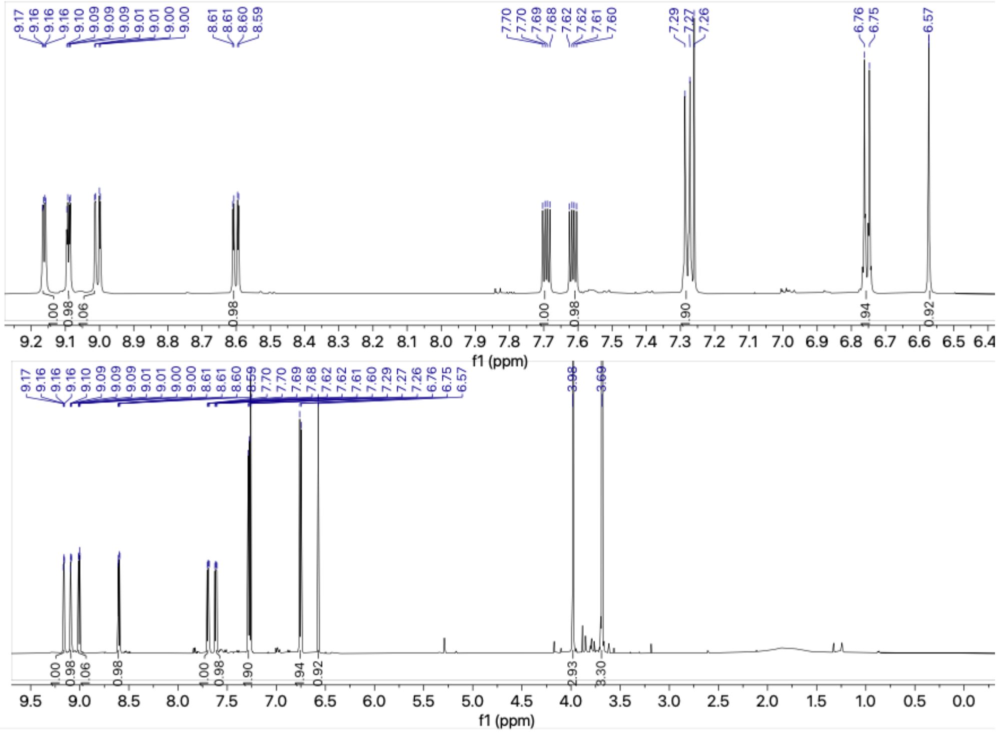

Figure S1: methyl 2-(4-methoxyphenyl)-2H-[1,4]oxazino[2,3-f][1,10]phenanthroline-3-carboxylate (PO1) ^{1}H NMR spectrum in $CDCl_3$.

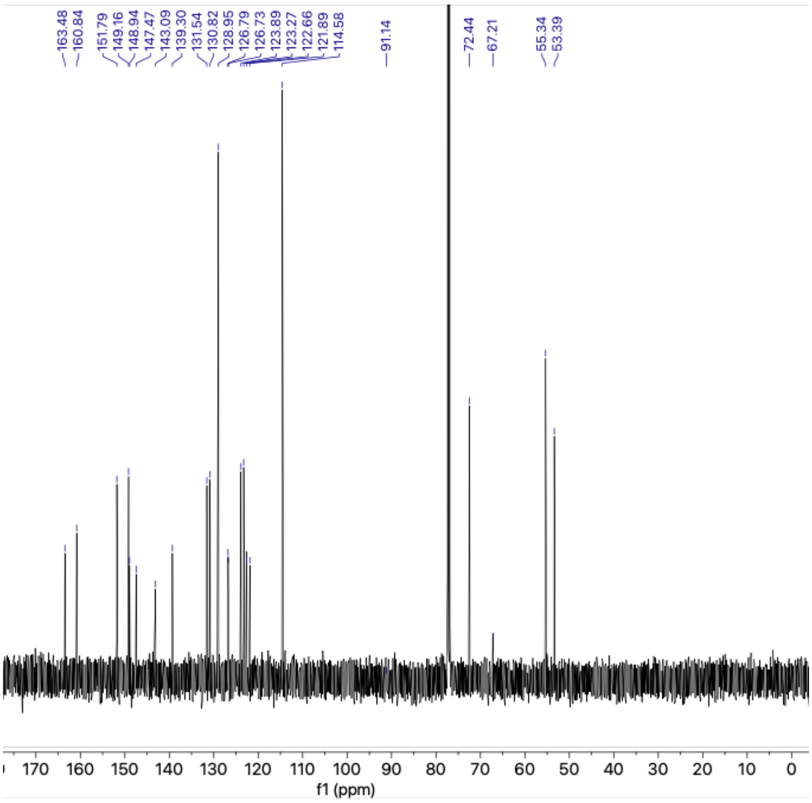

Figure S2: methyl 2-(4-methoxyphenyl)-2H-[1,4]oxazino[2,3-f][1,10]phenanthroline-3-carboxylate (PO1) ^{13}C NMR spectrum in $CDCl_3$.

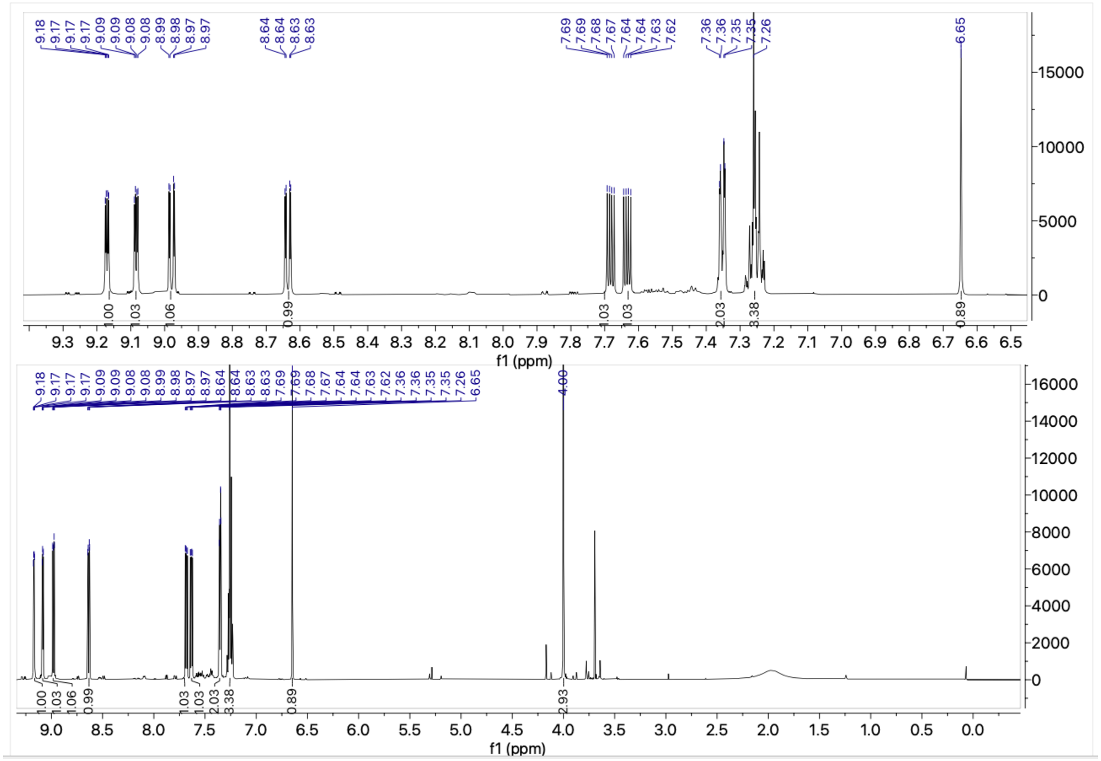

Figure S3: methyl 2-phenyl-2H-[1,4]oxazino[2,3-f][1,10]phenanthroline-3-carboxylate (PO2) ^{1}H NMR spectrum in $CDCl_3$.

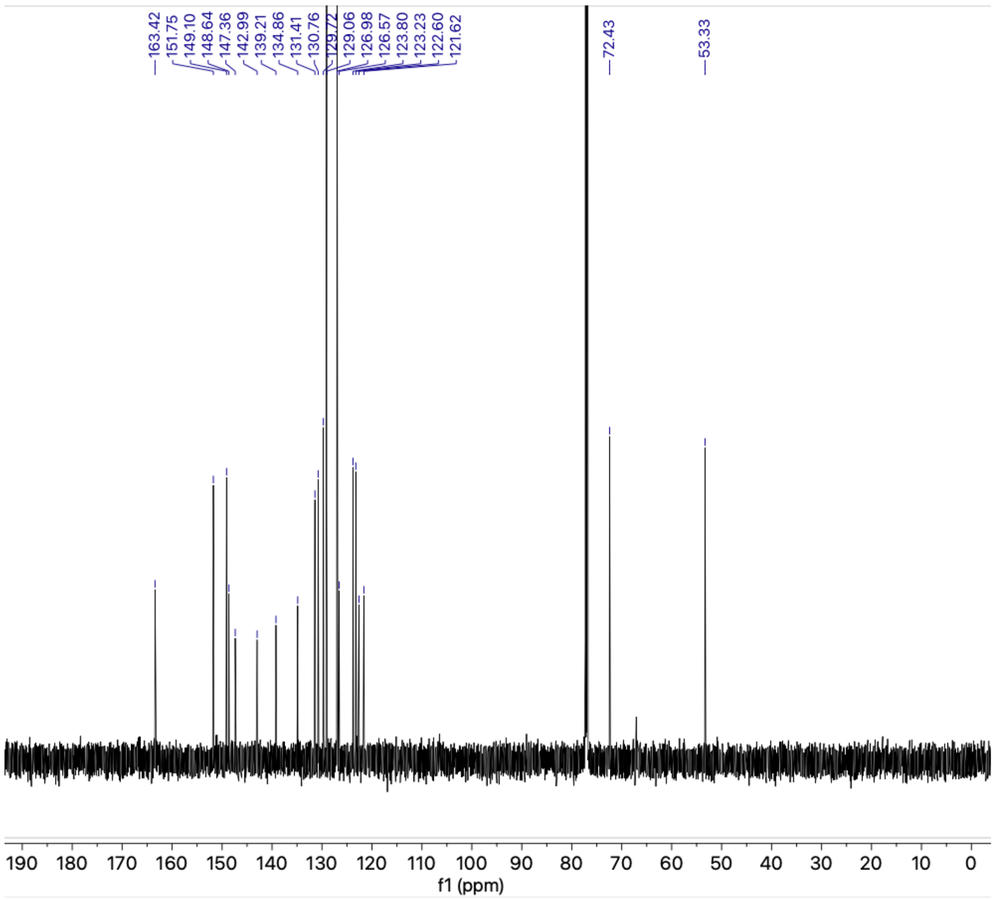

Figure S4: methyl 2-phenyl-2H-[1,4]oxazino[2,3-f][1,10]phenanthroline-3-carboxylate (PO2) ^{13}C NMR spectrum in $CDCl_3$.

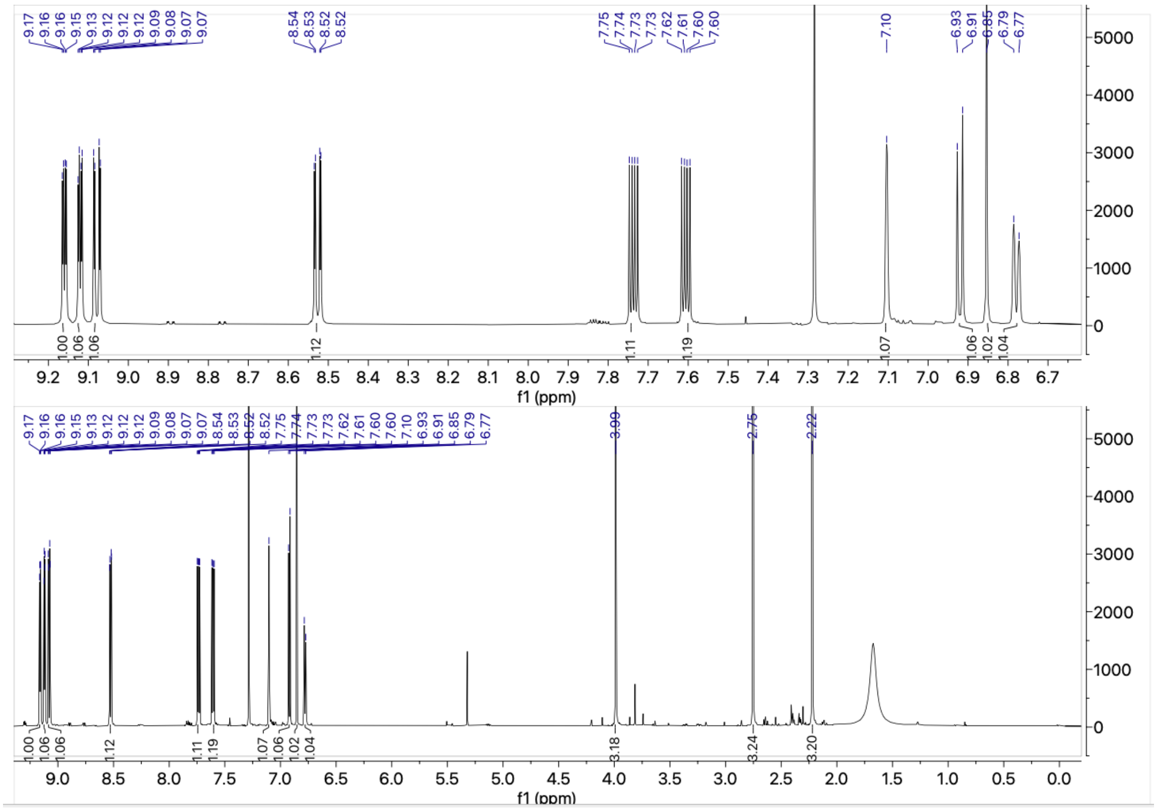

Figure S5: methyl 2-(2,4-dimethylphenyl)-2H-[1,4]oxazino[2,3-f][1,10]phenanthroline-3-carboxylate (PO3) ^{1}H NMR spectrum in $CDCl_3$.

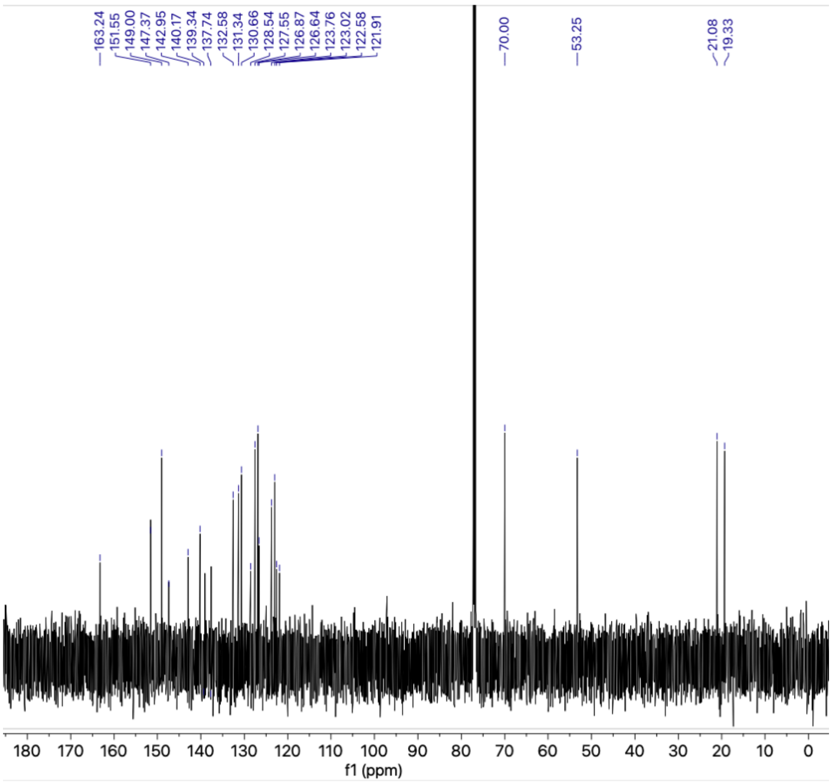

Figure S6: methyl 2-(2,4-dimethylphenyl)-2H-[1,4]oxazino[2,3-f][1,10]phenanthroline-3-carboxylate (PO3) ^{13}C NMR spectrum in $CDCl_3$.

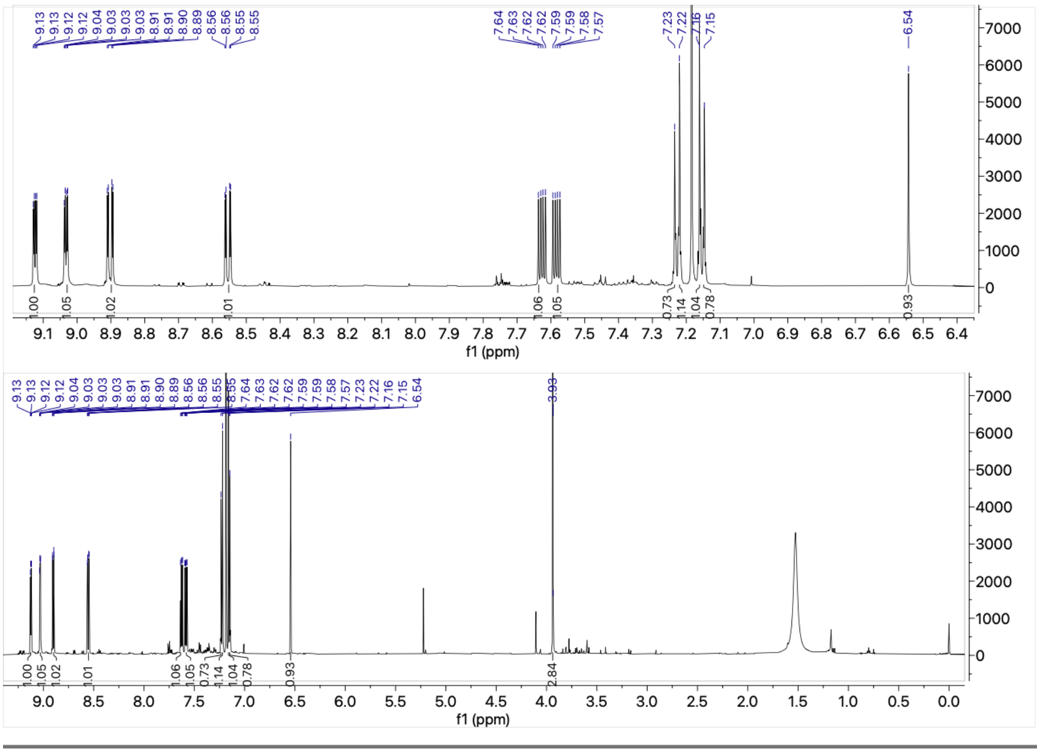

Figure S7: methyl 2-(4-chlorophenyl)-2H-[1,4]oxazino[2,3-f][1,10]phenanthroline-3-carboxylate (PO4) ^{1}H NMR spectrum in $CDCl_3$.

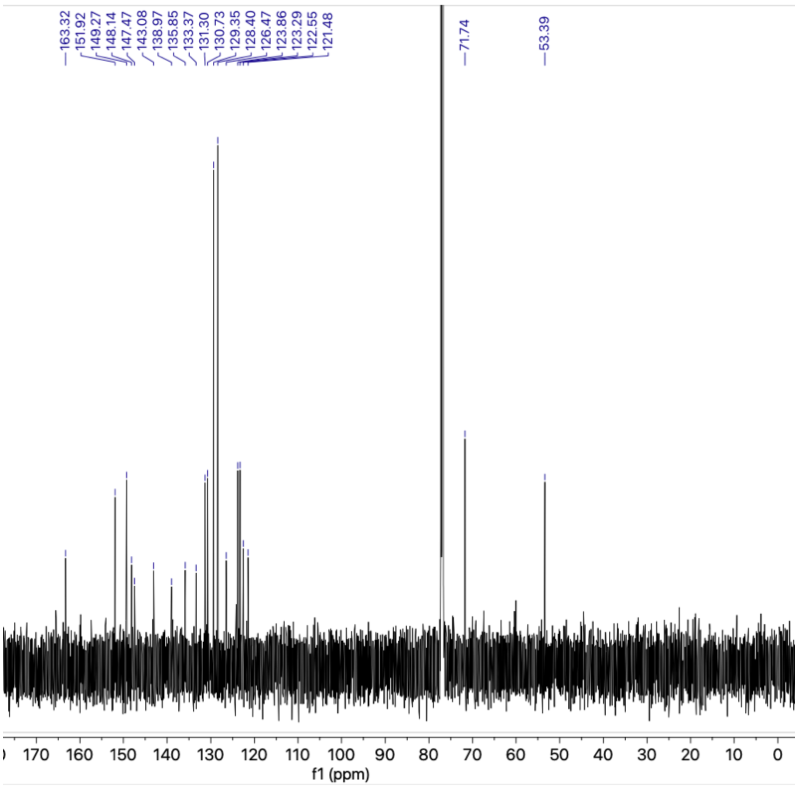

Figure S8: methyl 2-(4-chlorophenyl)-2H-[1,4]oxazino[2,3-f][1,10]phenanthroline-3-carboxylate (PO4) ^{13}C NMR spectrum in $CDCl_3$.

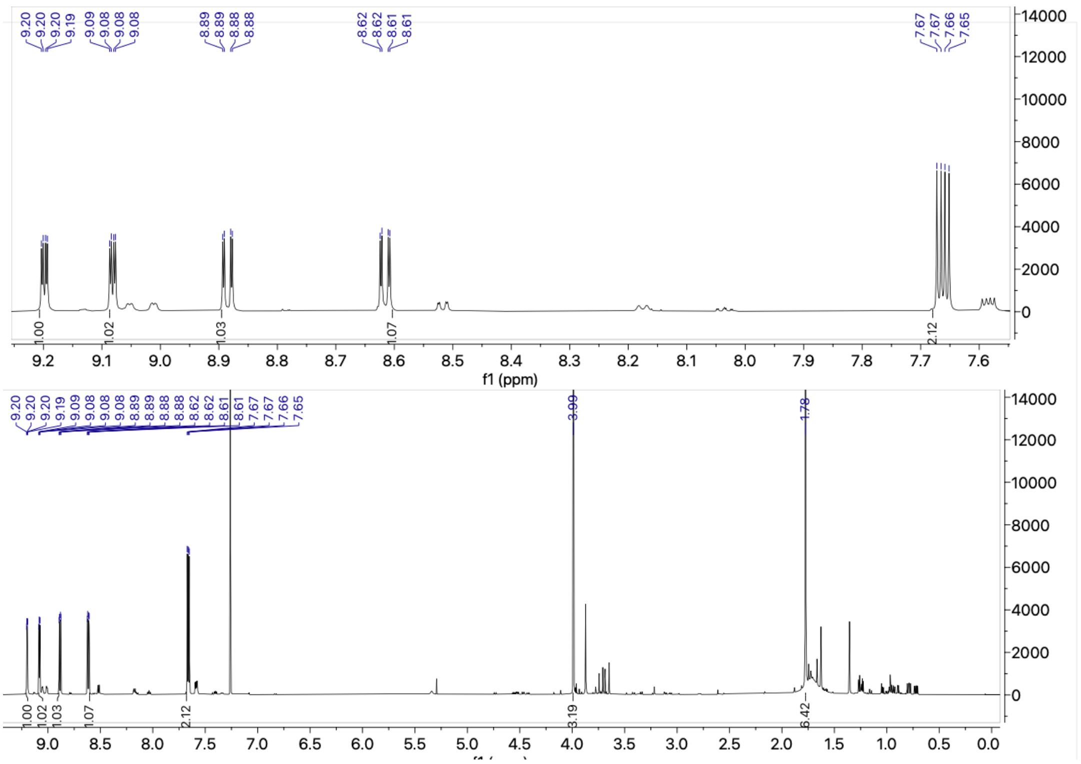

Figure S9: 2,2-dimethyl-2H-[1,4]oxazino[2,3-f][1,10]phenanthroline-3-carboxylate (PO5) ^{1}H NMR spectrum in $CDCl_3$.

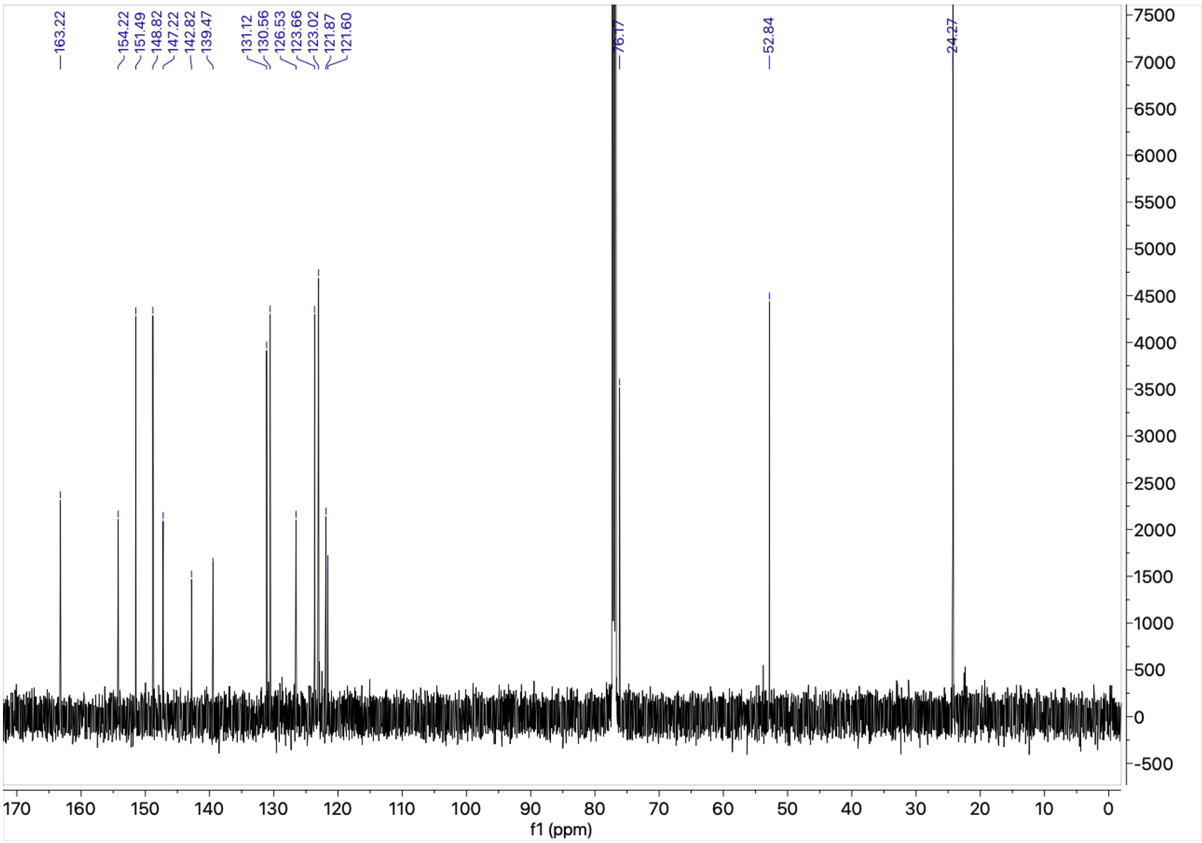

Figure S10: 2,2-dimethyl-2H-[1,4]oxazino[2,3-f][1,10]phenanthroline-3-carboxylate (PO5) ^{13}C NMR spectrum in $CDCl_3$.

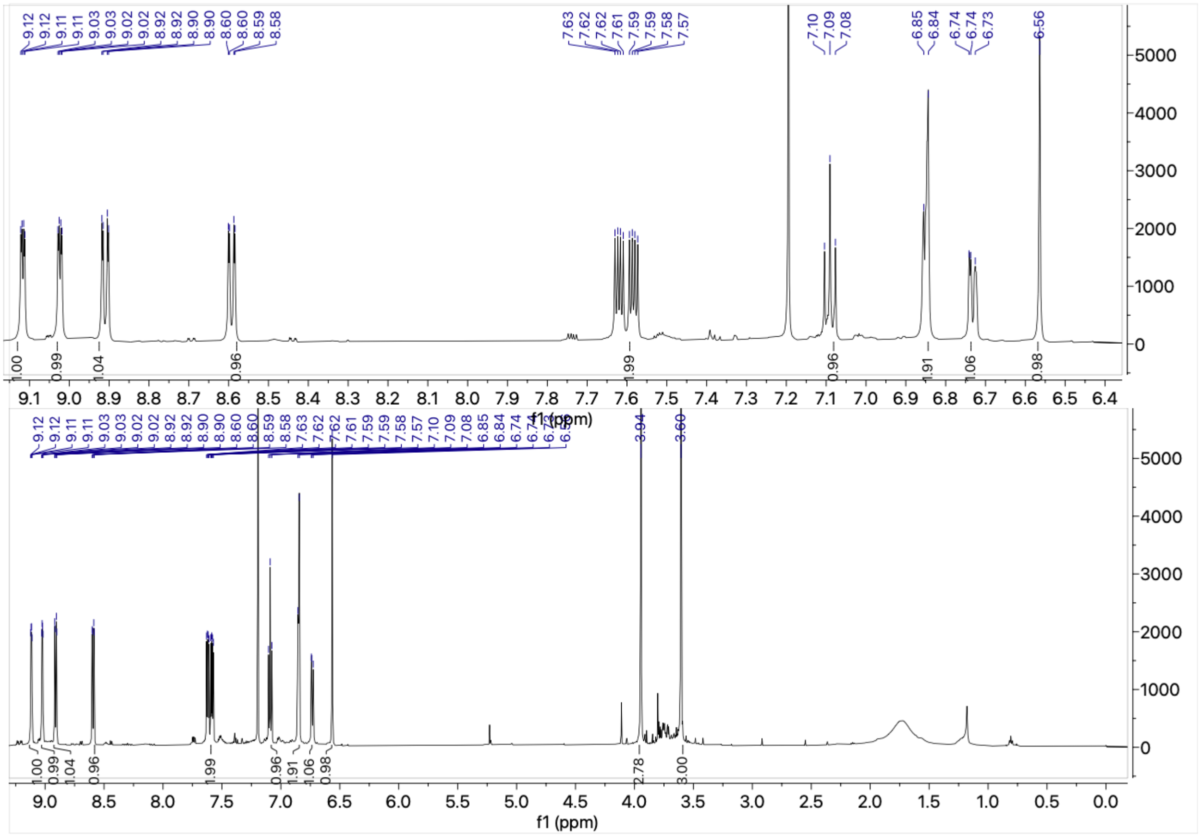

Figure S11: methyl 2-(3-methoxyphenyl)-2H-[1,4]oxazino[2,3-f][1,10]phenanthroline-3-carboxylate (PO6) ^{1}H NMR spectrum in $CDCl_3$.

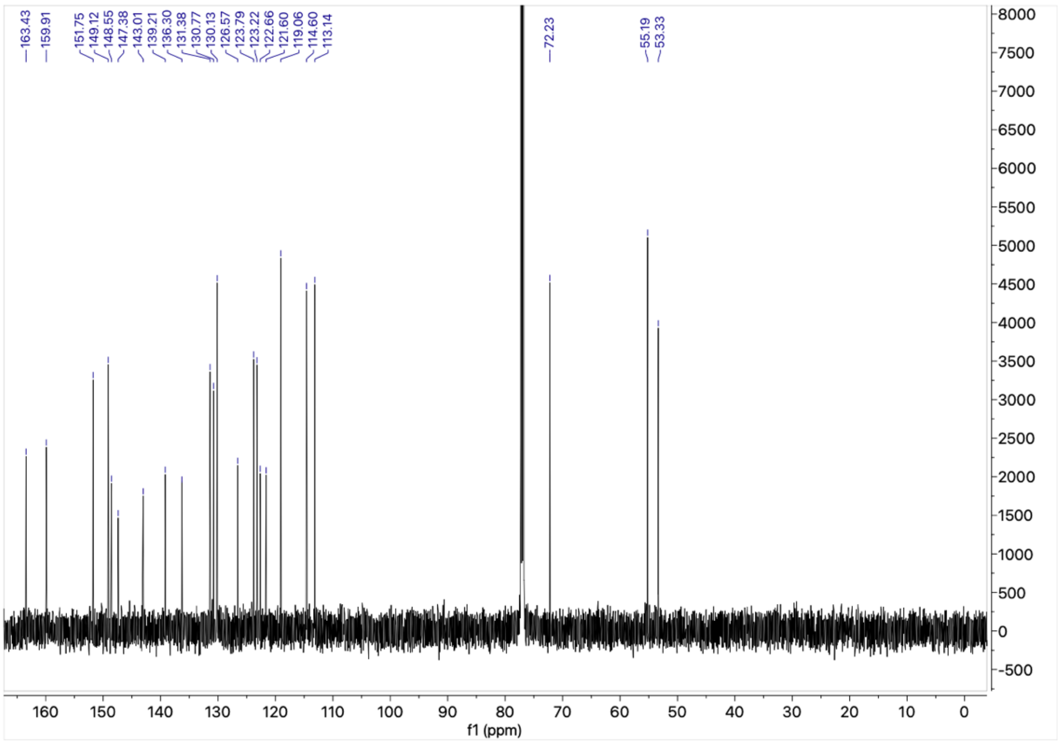

Figure S12: methyl 2-(3-methoxyphenyl)-2H-[1,4]oxazino[2,3-f][1,10]phenanthroline-3-carboxylate (PO6) ^{13}C NMR spectrum in $CDCl_3$.

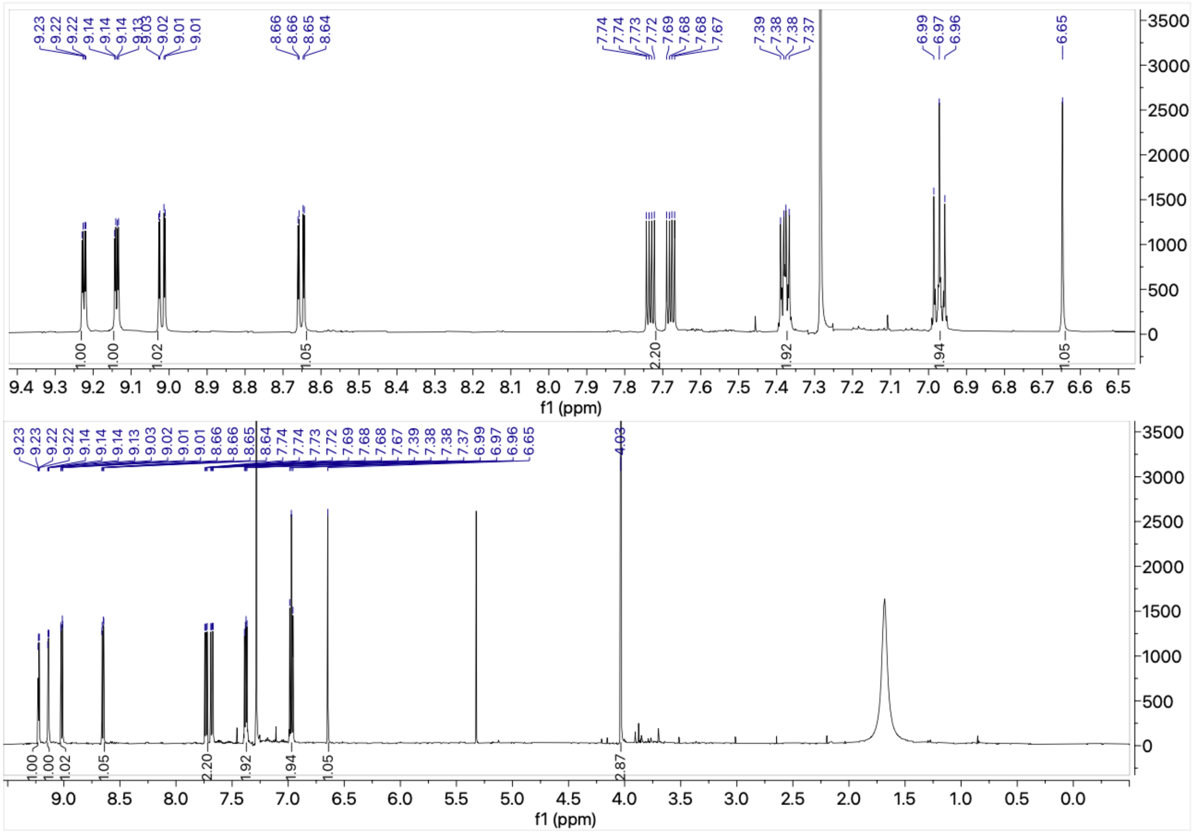

Figure S13: methyl 2-(4-fluorophenyl)-2H-[1,4]oxazino[2,3-f][1,10]phenanthroline-3-carboxylate (PO7) ^{1}H NMR spectrum in $CDCl_3$.

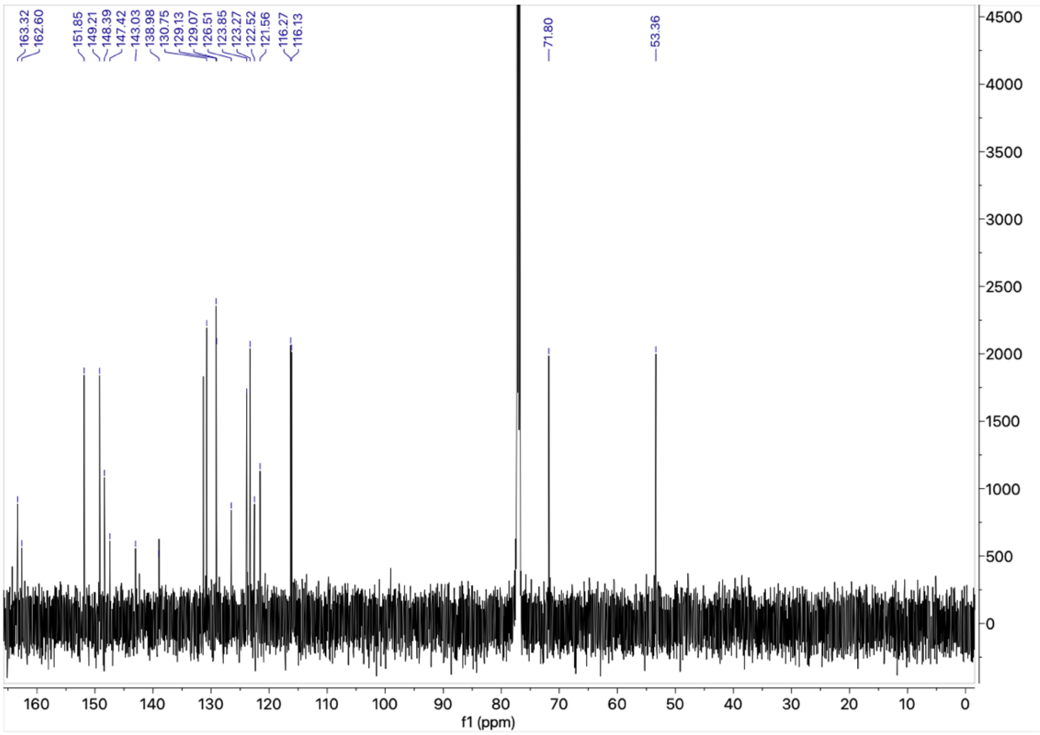

Figure S14: methyl 2-(4-fluorophenyl)-2H-[1,4]oxazino[2,3-f][1,10]phenanthroline-3-carboxylate (PO7) ^{13}C NMR spectrum in $CDCl_3$

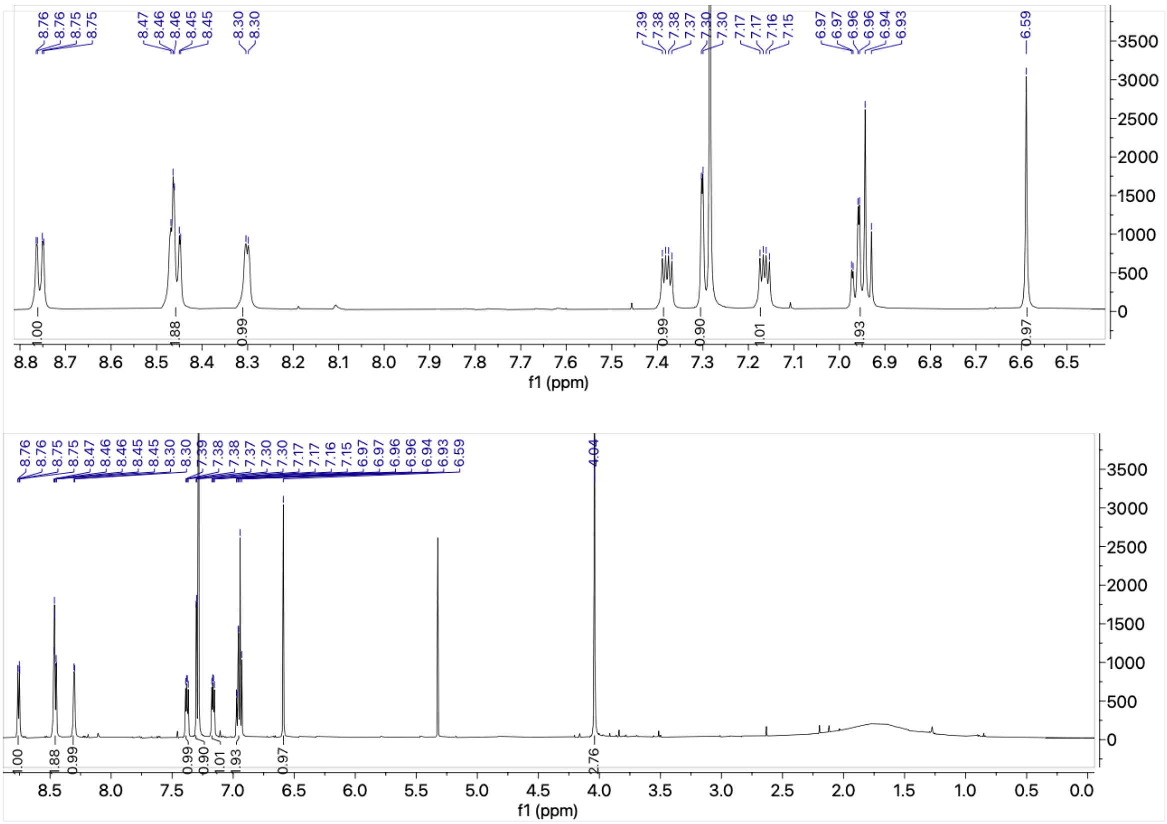

.

Figure S15: methyl 2-(3,4-dihydroxyphenyl)-2H-[1,4]oxazino[2,3-f][1,10]phenanthroline-3-carboxylate (PO8) ^{1}H NMR spectrum in $CDCl_3$.

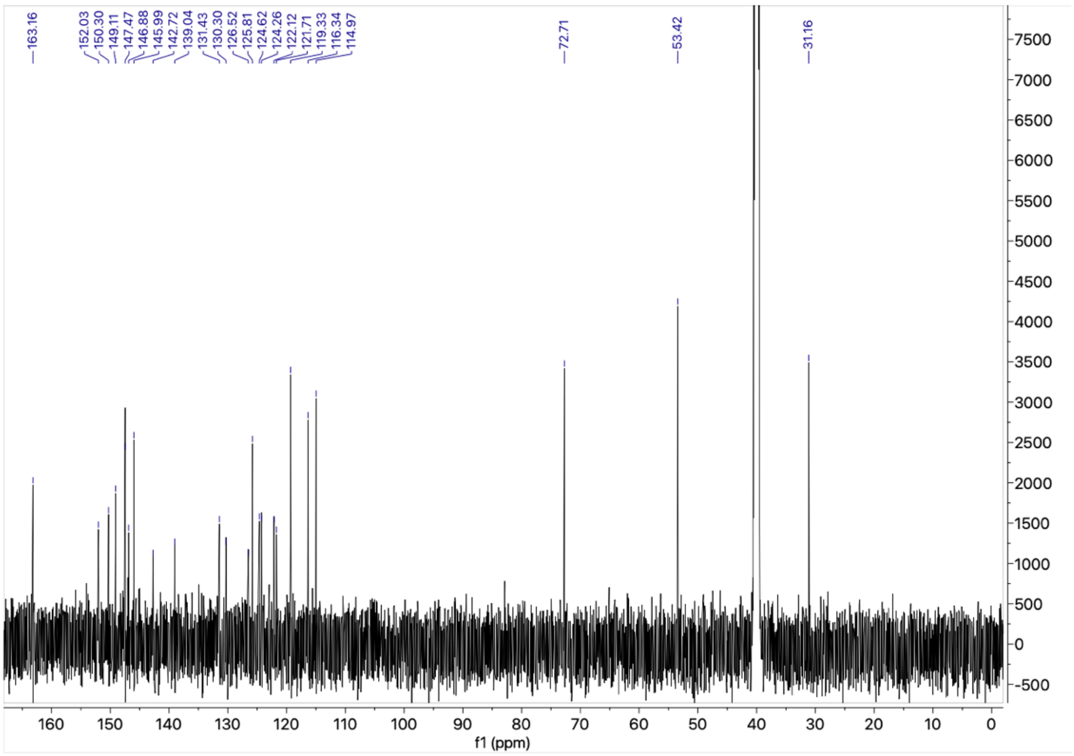

Figure S16: methyl 2-(3,4-dihydroxyphenyl)-2H-[1,4]oxazino[2,3-f][1,10]phenanthroline-3-carboxylate (PO8) ^{13}C NMR spectrum in $CDCl_3$.

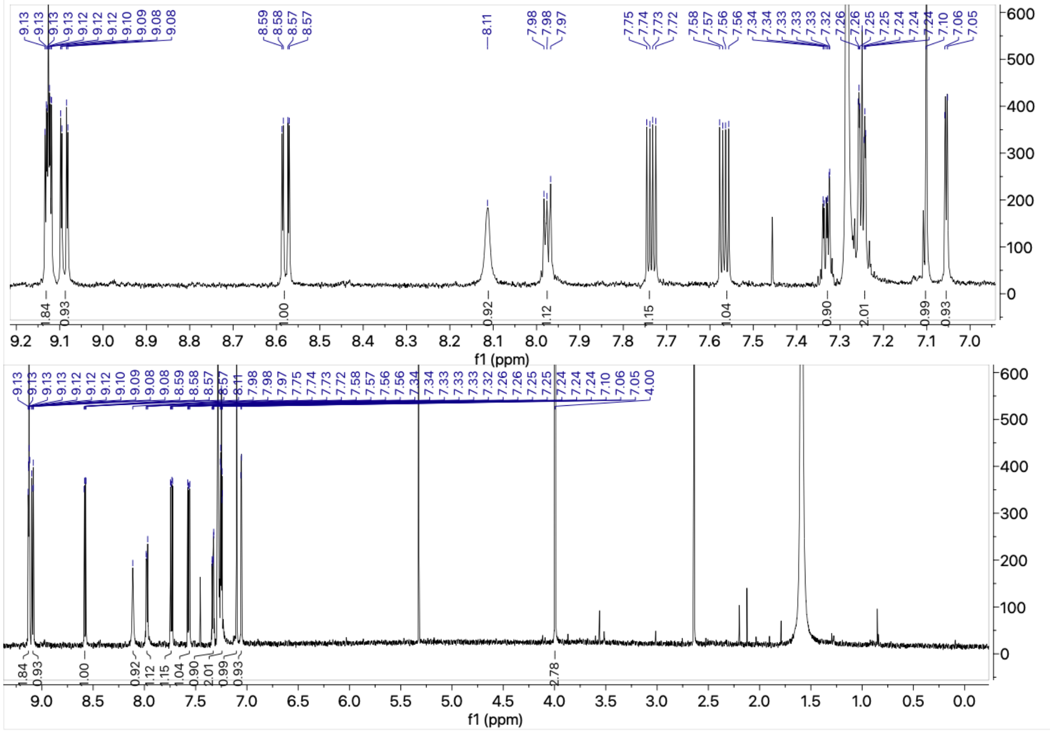

Figure S17: methyl 2-(1H-indol-3-yl)-2H-[1,4]oxazino[2,3-f][1,10]phenanthroline-3-carboxylate (PO9) ^{1}H NMR spectrum in $CDCl_3$.

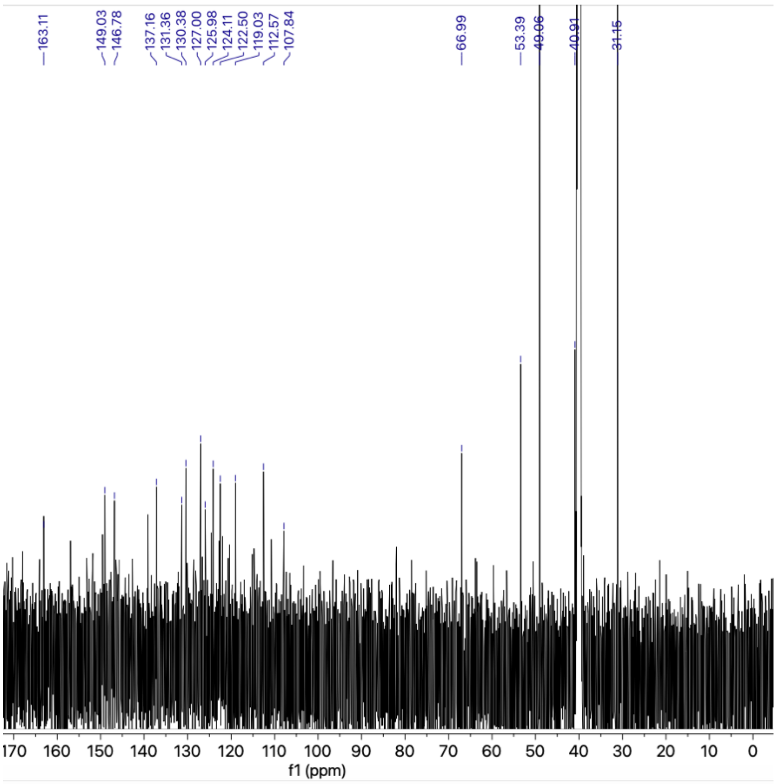

Figure S18: methyl 2-(1H-indol-3-yl)-2H-[1,4]oxazino[2,3-f][1,10]phenanthroline-3-carboxylate (PO9) ^{13}C NMR spectrum in $CDCl_3$.

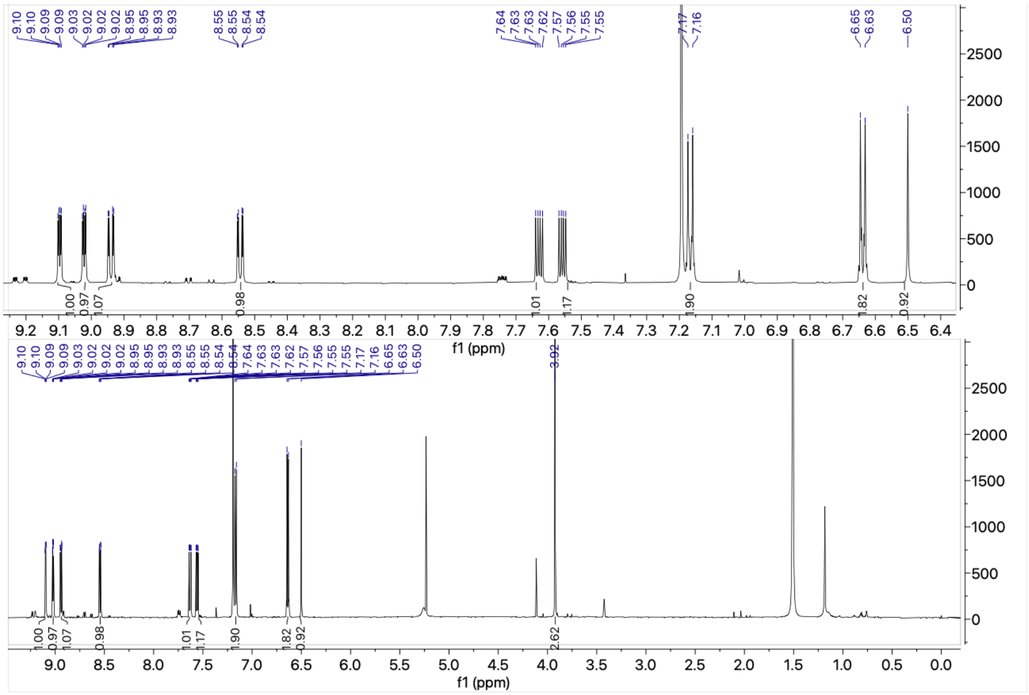

Figure S19: methyl 2-(4-hydroxyphenyl)-2H-[1,4]oxazino[2,3-f][1,10]phenanthroline-3-carboxylate (PO10) ^{1}H NMR spectrum in $CDCl_3$.

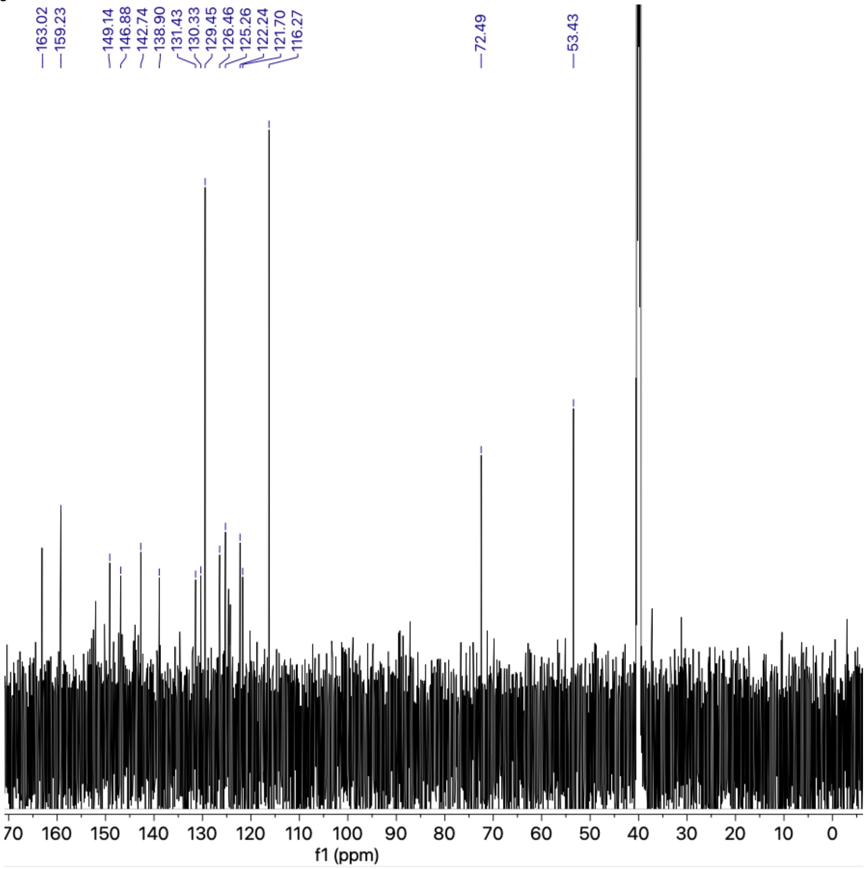

Figure S20: methyl 2-(4-hydroxyphenyl)-2H-[1,4]oxazino[2,3-f][1,10]phenanthroline-3-carboxylate (PO10) ^{13}C NMR spectrum in $CDCl_3$.

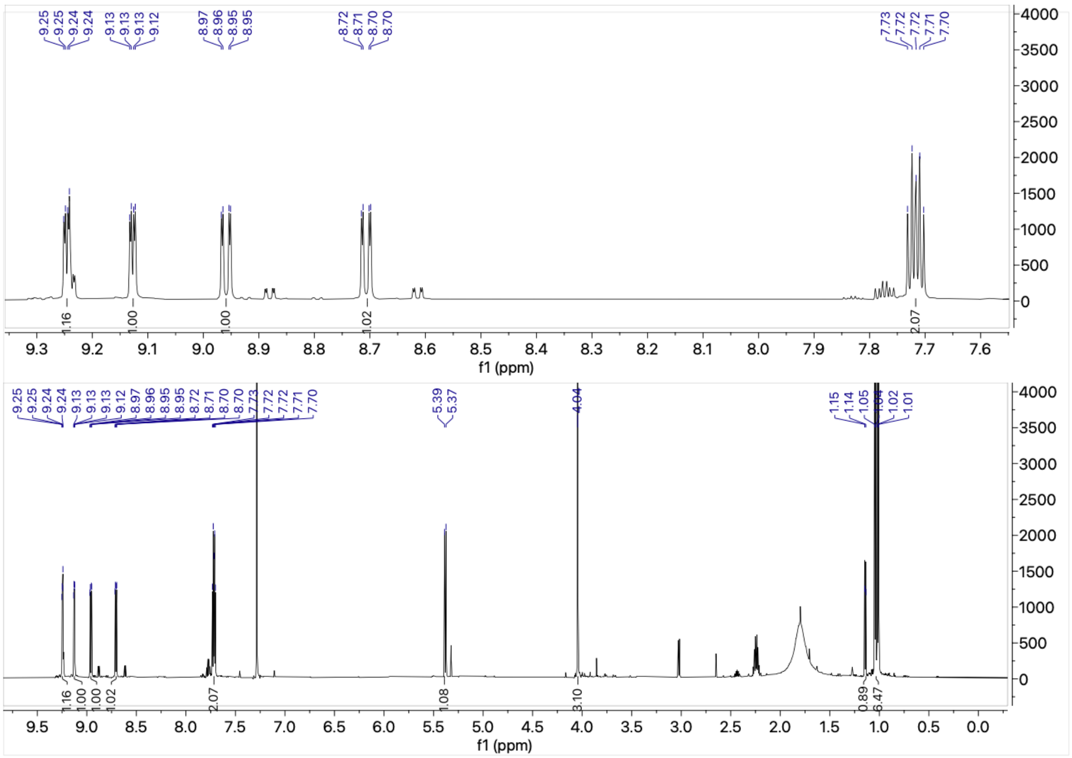

Figure S21: methyl 2-isopropyl-2H-[1,4]oxazino[2,3-f][1,10]phenanthroline-3-carboxylate (PO11) ^{1}H NMR spectrum in $CDCl_3$

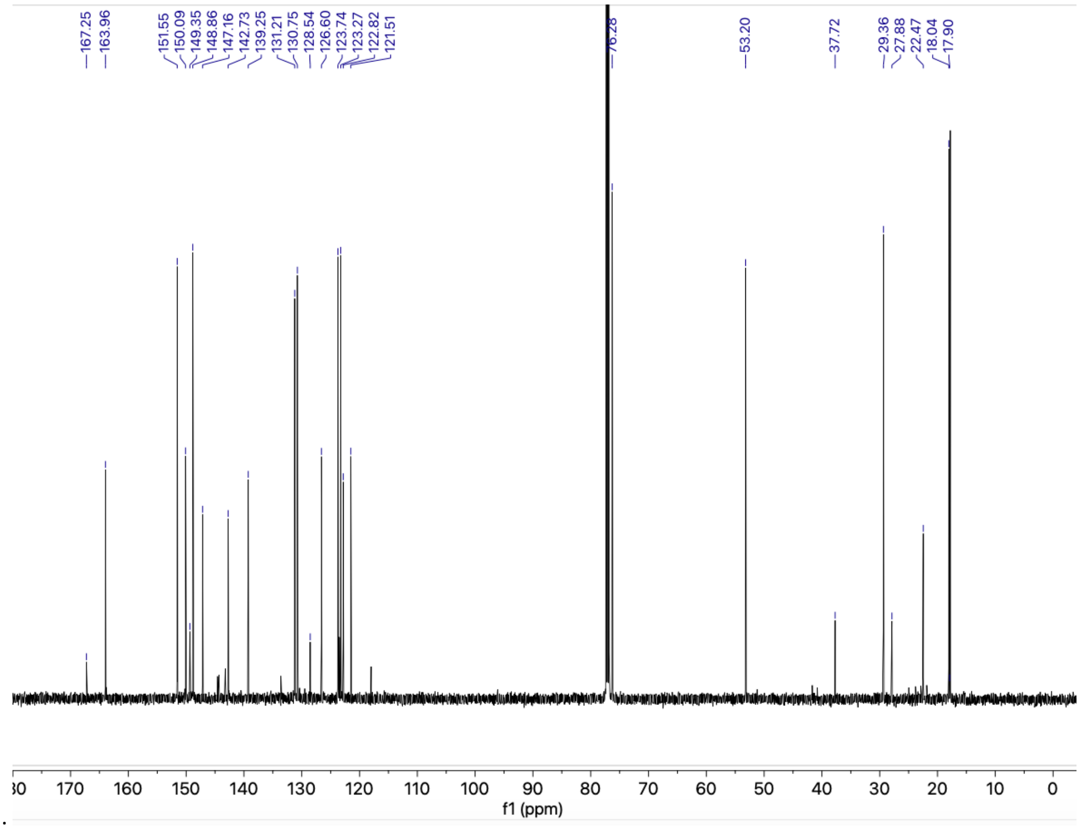

Figure S22: methyl 2-isopropyl-2H-[1,4]oxazino[2,3-f][1,10]phenanthroline-3-carboxylate (PO11) ^{13}C NMR spectrum in $CDCl_3$.

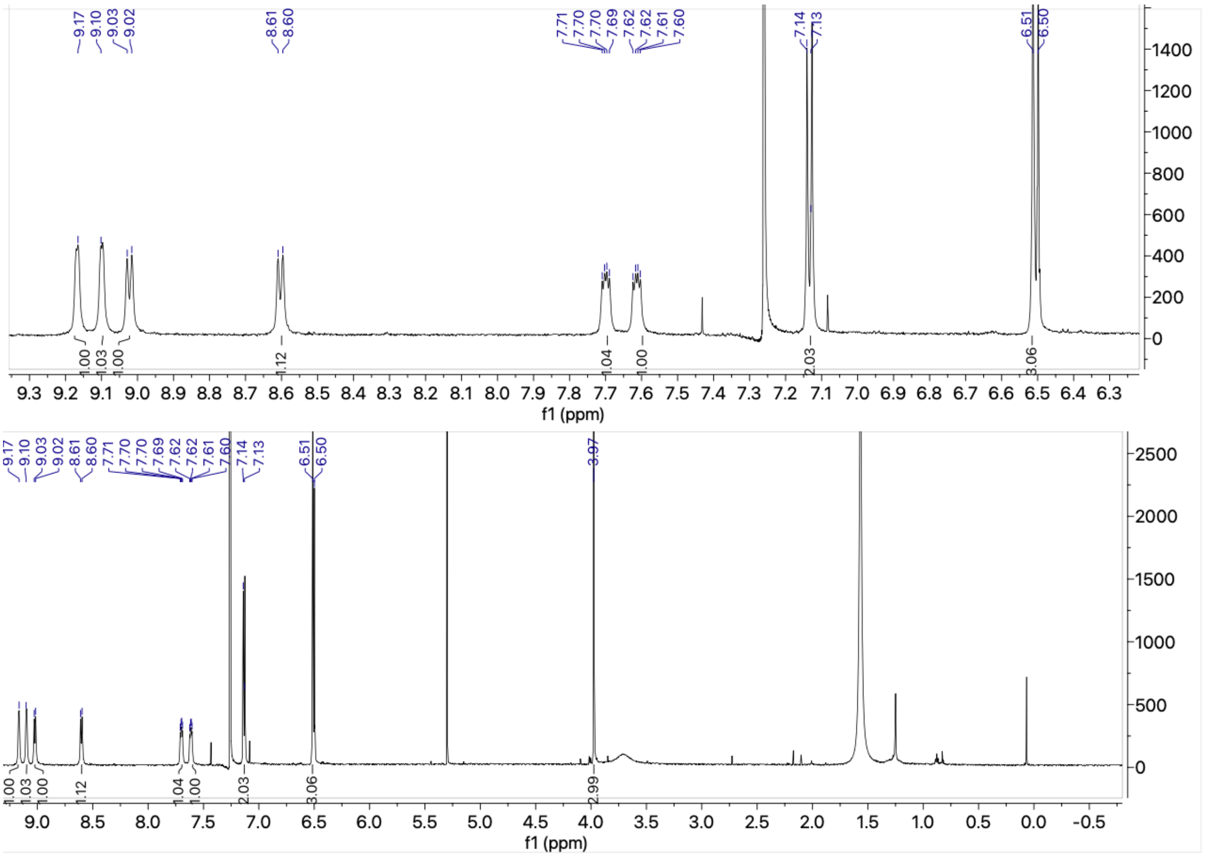

Figure S23: methyl 2-(4-aminophenyl)-2H-[1,4]oxazino[2,3-f][1,10]phenanthroline-3-carboxylate (PO12) ^{1}H NMR spectrum in $CDCl_3$.

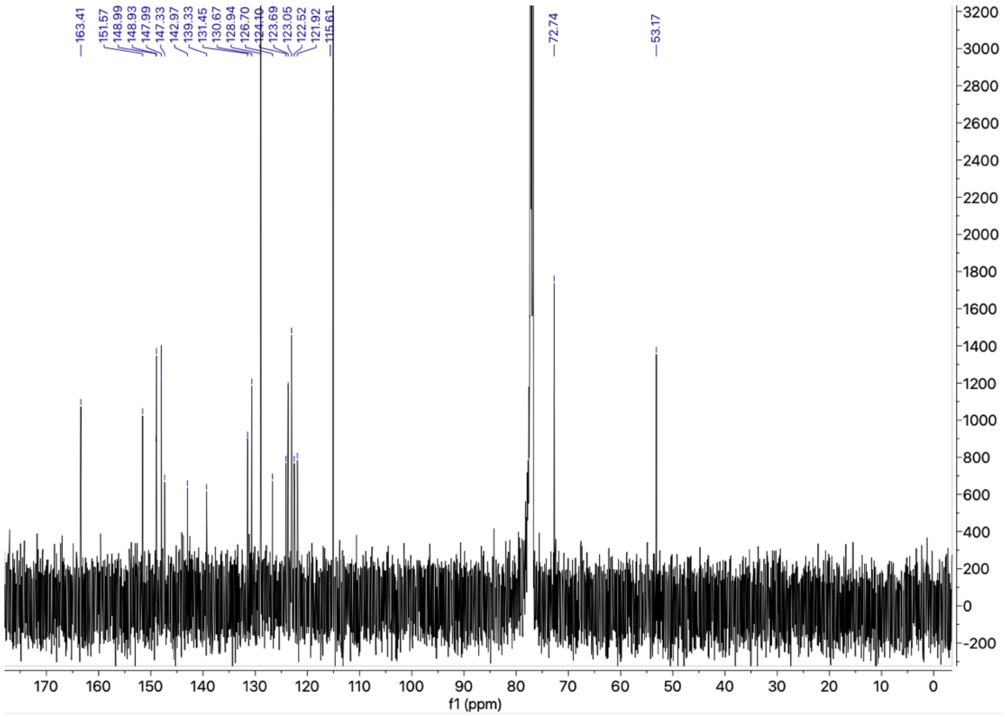

Figure S24: methyl 2-(4-aminophenyl)-2H-[1,4]oxazino[2,3-f][1,10]phenanthroline-3-carboxylate (PO12) ^{13}C NMR spectrum in $CDCl_3$.

S-3: TO Binding Constant Evaluation with CT DNA

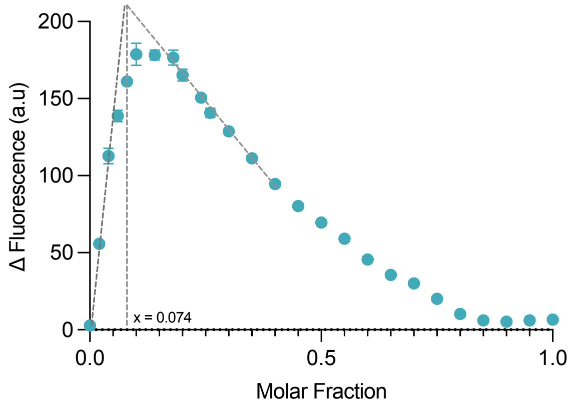

Figure S25: Job Plot of TO with CT DNA showing inflection point at molar ratio of 0.074, corresponding to a 13 bp binding site.

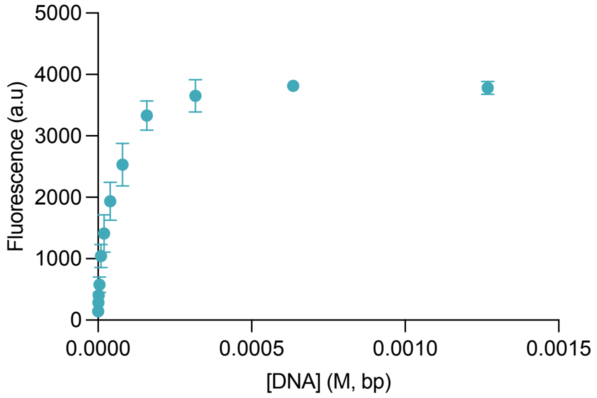

Figure S26: Plot of TO fluorescence intensity versus DNA concentration. All error bars indicate ± standard error and all experiements were run in triplicate (N=3).

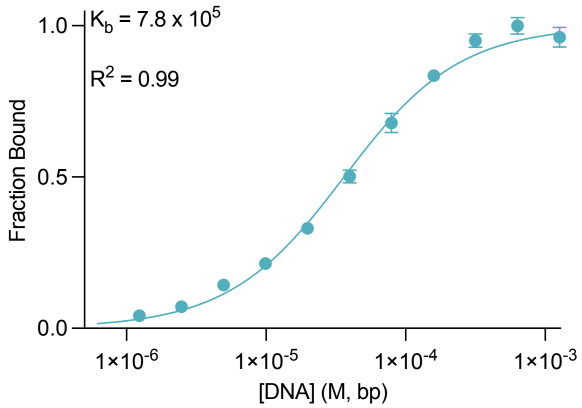

Figure S27: Plot of TO fraction bound vs DNA concentration extracted from Figure S26. The data was fitted with the Bard equation, using the 13 bp binding stoichiometry found from the Job plot to give K_b = 7.8 x 10^5 M^{-1} with R^2 = 0.99. All error bars indicate ± standard error and all experiements were run in triplicate (N=3).

S-4: Gel Electrophoresis

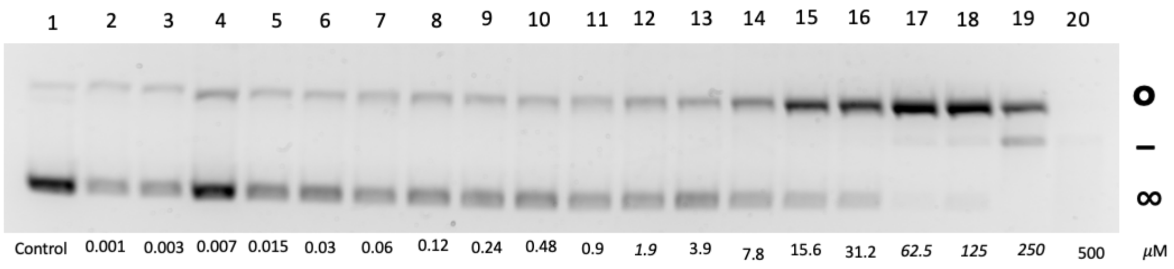

Figure S28: $Cu(ClO_4)_2$ cleavage gel. All lanes contained 400 ng of pUC19 DNA and 1 mM Na-*L* ascorbate. Control lane 1 contained no $Cu(ClO_4)_2$ while lanes 2-20 contained a concentration ramp of $Cu(ClO_4)_2$ prepared by 1:1 serial dilution.
